# Supplementary material for: Ice-sheet hydro-fracture not advanced inland by lower-elevation lake drainages in Kalaallit Nunaat
Source: Nat Commun. 2026 May 27;17:4598. doi: 10.1038/s41467-026-73033-z (PMC13216594; doi:10.1038/s41467-026-73033-z)
Supplement: Supplementary file 1 — Supplementary Information [file 41467_2026_73033_MOESM1_ESM.pdf]

Supplementary Information for

Ice-sheet hydro-fracture not advanced inland by lower-elevation lake drainages in Kalaallit Nunaat

Laura A. Stevens[1,2†], Meredith Nettles[3,4], Stacy Larochelle[3,5], Marianne Okal[6], Emily Falconer[1], Natalie Turner[1], Joshua Rines[7], Ching-Yao Lai[7], and George Lu[3]

[1]Department of Earth Sciences, University of Oxford, Oxford, UK
[2]Radcliffe Institute for Advanced Study at Harvard University, Cambridge, MA, USA
[3]Lamont-Doherty Earth Observatory, Palisades, NY, USA
[4]Department of Earth and Environmental Sciences, Columbia University, New York City, NY, USA
[5]Department of Earth, Planetary and Space Sciences, UCLA, Los Angeles, CA, USA
[6]EarthScope Consortium, Boulder, CO, USA
[7]Department of Geophysics, Stanford University, Stanford, CA, USA

[†]Correspondence should be addressed to laura.stevens@earth.ox.ac.uk

Supplementary Information

0. Physical plausibility of hydro-fracture event clusters (Text S0).
1. Chronology for hydro-fracture event cluster C1 (Text S1, Table S1, and Figures S1).
2. Chronology for hydro-fracture event cluster C2 (Text S2, Table S2, and Figures S2).
3. Chronology for hydro-fracture event cluster C3 (Text S3, Table S3, and Figures S3).
4. Chronology for hydro-fracture event cluster C4 (Text S4, Table S4, and Figures S4).
5. Chronology for hydro-fracture event cluster C5 (Text S5, Table S5, and Figures S5).
6. Chronology for hydro-fracture event cluster C6 (Text S6, Table S6, and Figures S6).
7. Table S7: Summary statistics of mechanistic lake-drainage catalogues.
8. Table S8. Size of physically plausible hydro-fracture and moulin-drainage event clusters.
9. Table S9: Subglacial flood propagation speeds.
10. Table S10. Station-pair choices for estimating vertical strain rate.
11. Table S11. Errors in bed-separation rate.

Text S0. Physical plausibility of event clustering

In this document, we interrogate whether the C1–6 temporal clusters of hydro-fracture events are plausible examples of inter-lake, hydro-fracture-event triggering using modeling considerations, Global Navigation Satellite System (GNSS) observations of ice-sheet surface positions, and pressure-logger observations of lake depth. First, we examine three different modeling considerations for physical plausibility: (1) the extent of high-tensile-stress (>200 kPa) regions produced by subglacial-blister opening and basal slip[1,2]; (2) the up-ice-flow extent of tensile-stress regions due to slippery patches[3]; and, (3) propagation rates and pathways of subglacial floods. Where possible, we ground truth these theoretical constraints using GNSS surface-position estimates that quantify strain-rate transients and bed separation produced by hydro-fracture events and travelling subglacial floods. Finally, for the lake pairs and triplets that hydro-fracture within our GNSS array, we present horizontal and vertical timeseries of 15-s-resolution ice-sheet surface deformation observations. We use these GNSS position observations—alongside observations of lake volume loss recorded by pressure loggers where available—to determine the drainage timing and sequencing of eleven GNSS-instrumented hydro-fracture events.

Text S0.1 Modeling considerations

Modeling consideration (1) estimates the spatial extent of high tensile stress produced by subglacial-blister opening and basal slip over elastic timescales of ice deformation (<1 day)[1,2]. We define a radial blister[2] of the maximum, FASTER[4]-estimated volume of the lake within the melt season. The height of this blister defines the amount of basal-cavity opening at a depth of the ice-sheet thickness within an elastic halfspace[1]. At this same depth within the halfspace, we impose 0.5 m of bed-parallel slip within the circumference of the blister in the down-ice-flow direction[1]. Ice-sheet surface deformation due to this prescribed basal-cavity opening and slip is forward modelled using the Okada (1985) Green functions[5], and the modelled surface-strain components are presented as maximum principal stress σ_1 using mid-range material properties for glacial ice of a Poisson ratio of 0.3 and a shear modulus of 1.5 GPa[6]. We use a sign convention that $\sigma > 0$ equals tension.

Modeling consideration (2) estimates the average surface tensile stress produced by slippery ice-sheet bed patches during the days following the hydro-fracture event. We assume each hydro-fracture event creates an along-ice-flow slippery-patch with a length scale l equal to twice the idealized-blister diameter. This assumption likely produces a maximum estimate of the actual water-lubricated spatial extent, given that the idealized-blister diameter is calculated from a lake's observed maximum volume. Following ref. [3], the perturbed along-flow, tensile ice-sheet surface stress at the inland boundary of the slippery patch can be estimated through $\rho g l \alpha / 4$, where ρ is ice density (917 kg m^{3}), g is gravitational acceleration (9.81 m s^{-2}), and α is the ice-sheet surface slope over the length of the slippery patch[3]. For the 39 in-cluster hydro-fracture events, the average slippery-patch length l is 8.4 km and the average ice-sheet surface slope α across these slippery patches is 0.006, yielding a representative stress value at this inland boundary of $+113$ kPa. This estimate uses the depth-averaged shallow ice stream approximation and has been validated against simulations that use Stokes flow[3]. These viscous stresses will exponentially decay moving inland of the slippery patch[3], and we use the average slippery-patch length for the 39 in-cluster hydro-fracture events, alongside the observational constraints provided by the mechanistic lake-drainage catalogues, to guide our analyses of which additional hydro-fracture and moulin-lake drainage events may fall inside of perturbed-stress regions created by slippery ice-sheet bed patches.

For modeling consideration (3) of observed[7,8] and modelled[9] propagation rates and pathways of traveling subglacial blisters, we estimate the locations of subglacial-flood-propagation fronts caused by hydro-fracture events using GNSS observations of the speed of three different travelling subglacial

blisters within our array (Table S9). Observed propagation speeds range from 0.33–0.45 m s^{-1}; fit within the observed, expected range for ice-sheet supraglacial lake drainages[8]; and are slightly faster than the ~0.3 m s^{-1} (~26 km d^{-1}) speed reported for a Paakitsoq region lake drainage[7]. We settle on a value of 0.4 m s^{-1} (35 km d^{-1}) for our modelled subglacial-flood-propagation fronts. We take the potential blister-propagation pathway to encompass both down-ice and down-subglacial-pathway orientations because—for many regions in our study area—subglacial-drainage pathways develop at high angle to ice-flow direction due to basal ridges running in the across-ice-flow direction[10,11]. Finally, while we consider all hydro-fracture and moulin-drainage events encompassed within the flood-propagation front as plausible examples of inter-event triggering, high-pressure subglacial blisters shed volume to the surrounding drainage system as they travel[9], causing blisters to lose height[8] and reducing their capacity to induce tensile ice-surface stresses through ice-sheet uplift[1]. Thus, while a velocity response can be observed without corresponding ice-sheet uplift[8] during latter-stage propagating floods, these events may be less likely to drive large, tensile ice-surface stresses[1].

Text S0.2 GNSS-derived quantities

We ground truth theoretical constraints using GNSS surface-position estimates that quantify strain-rate transients and bed separation produced by hydro-fracture events and travelling subglacial floods. Following ref. [1], 30-min-resolution horizontal strain rates between GNSS stations ($\dot{\varepsilon}_{xx}$, $\dot{\varepsilon}_{yy}$) are calculated from surface-position estimates and 30-min-resolution velocities in the along- and across-flow directions (Figs. S0.1, S0.2). For each station we estimate a bed separation rate $\dot{c}$, which is the component of the ice-sheet surface vertical velocity w_s remaining after subtracting vertical motion due the ice sheet's advective, bed-parallel flow and its thickness-integrated vertical strain rate[12–14]. Bed separation rate $\dot{c}$ is often invoked as a proxy for subglacial cavity opening and/or sediment dilation and is estimated following:

$$\dot{c} = w_s - u_b \tan\theta_b - \dot{\varepsilon}_{zz} H \quad , \quad (1)$$

where u_b is horizontal sliding at the ice-bed interface, θ_b is the local bed slope, $\dot{\varepsilon}_{zz}$ is vertical strain rate, and H is ice-sheet thickness[12]. Ice-sheet surface vertical velocity w_s is estimated using sliding least-squares regression (Methods). Ice-sheet thickness H is taken to be the ice thickness at the nearest location in the 150-m-horizontal-resolution BedMachine Greenland v.5 thickness product[15,16] (Table S11).

The bed-parallel component of vertical flow ($u_b \tan\theta_b$) is estimated assuming a bed separation rate of zero and constant vertical strain and bed-parallel motion during the pre-runoff time periods, allowing for Eq. 1 to be expressed as:

$$u_{b,Spring} \tan\theta_b = w_{s,Spring} - \dot{\varepsilon}_{zz,Spring} H \quad , \quad (2)$$

where subscripts "Spring" indicate mean values calculated over 2022/150–160 or 2023/150–160. Aligned with previous workers[12–14], for the purposes of this study's questions we further assume (1) that ice-sheet surface and basal velocities are equivalent, and (2) that vertical strain rate is depth invariant. Such assumptions allow for $u_{b,Spring}$ to be estimated as stations' mean surface velocity $u_{s,Spring}$ over the pre-runoff time periods; for local bed slope θ_b to be estimated as the slope of a 1.65–by–1.65 km region consisting of eleven BedMachine 150-m bed-elevation estimates[15,16] that is then projected in the direction of $u_{s,Spring}$; and for $\dot{\varepsilon}_{zz,Spring}$ to be calculated as a residual from Eq. 2.

Returning to Eq. 1, the bed-parallel component of vertical flow ($u_b \tan\theta_b$) during the melt season is estimated using a constant local bed slope θ_b and by assuming that along-flow surface velocities u_s are equivalent to u_b. The vertical strain rate $\dot{\varepsilon}_{zz}$ is calculated from horizontal strain rates ($\dot{\varepsilon}_{xx}$, $\dot{\varepsilon}_{yy}$) between station pairs, assuming ice is incompressible (i.e., $\dot{\varepsilon}_{zz} = -(\dot{\varepsilon}_{xx} + \dot{\varepsilon}_{yy})$)[12]. Following this approach, the estimate of bed separation rate $\dot{c}$ at a specific station requires making station-pair choices (Table S10) for estimating horizontal strain rates that then dictate the estimation of

the thickness-integrated vertical strain rate term $\dot{\varepsilon}_{zz}H$ (Eq. 1). For stations farther than a few kilometers away from draining lake(s), station-pair choices are made to reduce the influence of short-time-period, lake-drainage-sourced horizontal strain-rate transients on $\dot{c}$ estimates; however, reduction in station density near MLOW, MHIH, and QIET necessitates the use of station-pair combinations that include stations within the lake clusters. In these instances, care is taken to select a relevant lake-cluster station with the smallest lake-drainage-sourced deformation.

Estimated 1σ errors in bed-separation rates $\delta\dot{c}$ are calculated via arithmetic error propagation through Eq. 1, using error estimates of station horizontal and vertical velocities, station positions, ice-sheet thicknesses beneath stations, and bed slopes beneath stations (Table S11). Estimates of $\delta\dot{c}$ for different stations range from 0.003 to 0.021 m d^{-1}, when averaged in time from 2022/165–230 and from 2023/165–230 (Table S11). We take $\dot{c}$ perturbations three times greater than the upper end of our $\delta\dot{c}$ estimates (0.021 m d^{-1}) to be detectable given measurement precision, placing the bound on a detectable $\dot{c}$ perturbation at ± 0.06 m d^{-1} for the dataset. We present $\pm 3\sigma$ error envelopes for $\dot{c}$ timeseries on all relevant figures (Figs. S1.2, S2.2, S3.2, S4.2, S5.2, and S6.2).

Finally, for the lake pairs and triplets that hydro-fracture within our GNSS array, we present horizontal and vertical timeseries of 15-s-resolution ice-sheet surface deformation observations. The positions are presented without temporal smoothing, but with outliers further than 3σ from the mean within a moving, centred, 6-hr-width window removed.

Text S0.3 Lake discharge

We deployed RBR solo3 pressure loggers in lake basins to log continuously at 5-s resolution from late May through mid-September in each melt season. These pressure loggers successfully recorded lake depth during three hydro-fracture events: the L1A 2022 event, the L2A 2023 event, and the L2B 2023 event. We use these lake-depth observations to quantify lake discharge Q_{lake} during hydro-fracture events, providing an independent observation of the drainage timing and sequencing of the GNSS-instrumented hydro-fracture events.

Throughout the GNSS deployment, atmospheric pressure P_{air} was observed continuously at 30-s resolution by RBR DR-1050 pressure loggers deployed within the receiver boxes of GNSS stations G103 and G306. To estimate P_{air} above the lake-pressure loggers, we regress elevation against P_{air} observations at G103 and G306, and calculate estimated P_{air} at the elevation of the lake-pressure loggers. We subtract this estimated P_{air} at the elevation of the lake-pressure loggers from down-sampled, 30-s-resolution pressure recordings by the lake-pressure loggers to obtain timeseries of pressure due to water depth P_w. Water pressure P_w is converted to water depth d above the logger through $d = P_w/\rho_w g$, where ρ_w is the density of water (1000 kg m^{-3}). For all three lake-pressure loggers, a temporally consistent offset of ~1000 Pa is observed between the modelled atmospheric-pressure estimates at the elevation of the lake-pressure loggers and these loggers' observed pressure recordings prior to logger inundation. We take this offset to be a useful estimate of the likely observational error in our lake-depth observations: ±0.1 m.

We combine the timeseries of water depth above the logger d with the elevation of the lake-pressure-logger location in 2-m-resolution Digital Elevation Models (DEMs) of post-drainage basins created by the Polar Geospatial Center from Maxar imagery to estimate lake-surface elevation. The DEM of the post-drainage L1A basin was obtained on 2022/240; the DEM of the post-drainage L2A and L2B basins was obtained on 2022/225. We multiply the estimated, 30-s-resolution water depth of submerged DEM tiles by the 4-m^2-area of individual tiles to estimate lake volume, and differentiate this volume estimate to obtain 10-min-resolution estimates of lake discharge Q_{lake}. We use a sign convention that $Q_{lake} < 0$ equals water draining out of the lake. Our sole use of the pressure-logger observations in this work is to discern the sequencing of neighbouring hydro-fracture events. Thus, we do not adjust the FASTER-estimated maximum lake volumes used for the subglacial-blister and slippery-patch theoretical constraints for the three lakes instrumented with pressure loggers.

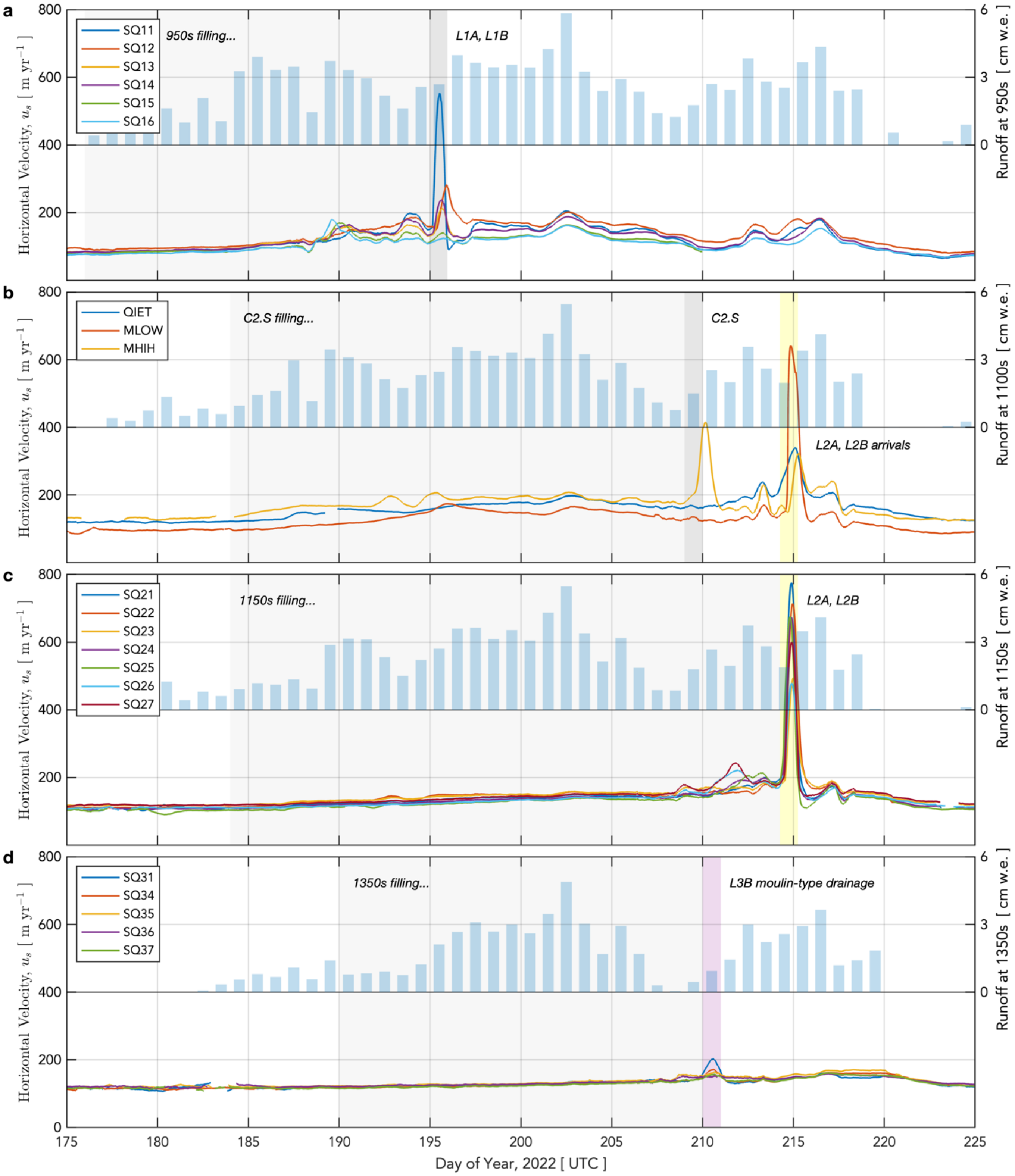

Figure S0.1. Horizontal velocities in 2022. (left *y*-axis) Horizontal velocity of GNSS stations grouped at (**a**) 950, (**b**) 1100, (**c**) 1150, and (**d**) 1350 m a.s.l. Daily runoff[17] (blue bars, right *y*-axis) shown for each station group. Light-grey shading shows timing of lake presence. Individual-day shading shows days of hydro-fracture events of lakes within the GNSS array. Velocities are calculated using a sliding least-squares window width of 36 hr, except for 2022/187–199 at the 950s, 2022/206–217 at the 1150s, 2022/206–212 at the 1350s, 2022/212–215 at MLOW, and 2022/206–212 at MHIH, wherein a sliding least-squares window width of 18 hr is used.

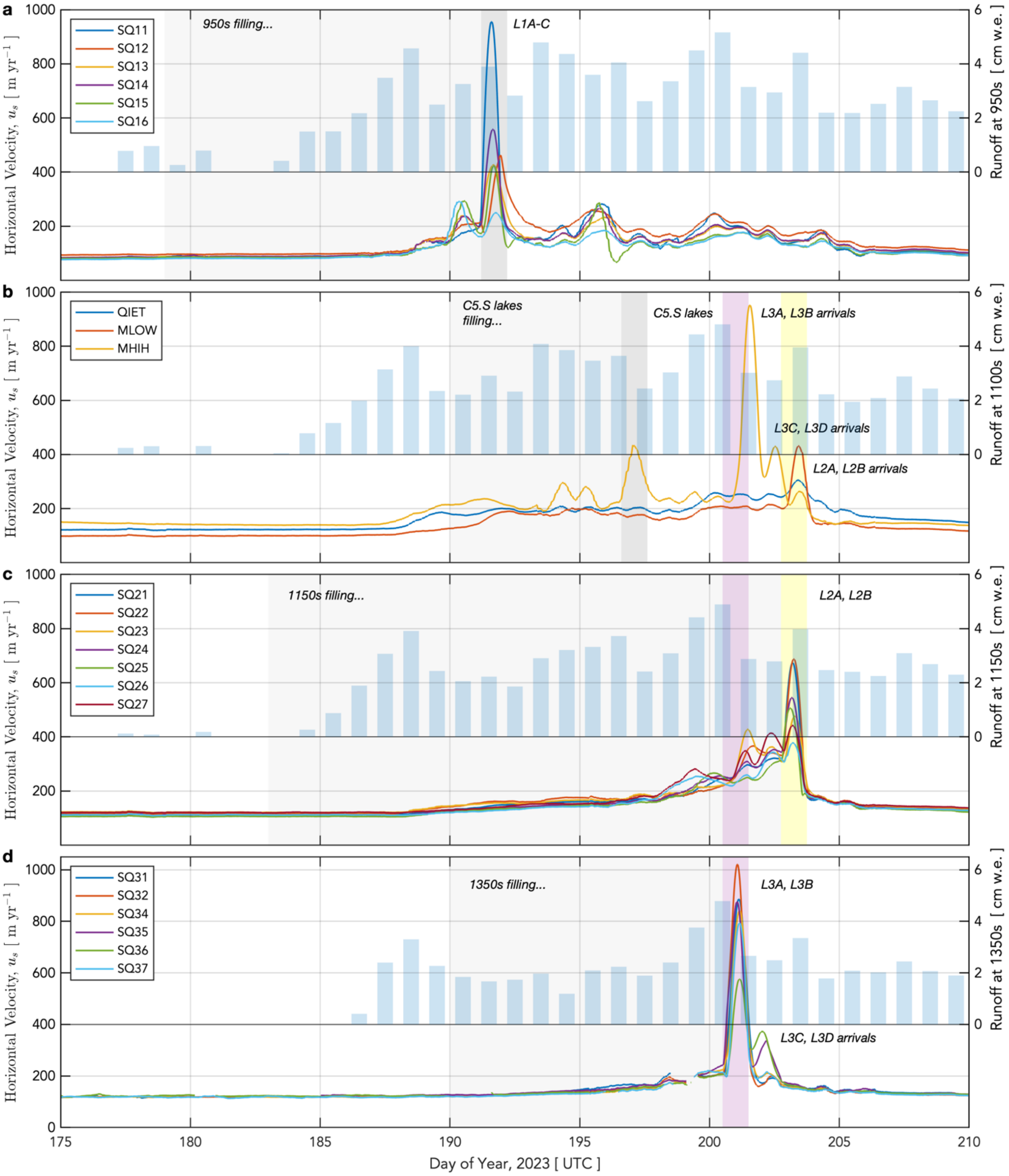

Figure S0.2. Horizontal velocities in 2023. (left y-axis) Horizontal velocity of GNSS stations grouped at (**a**) 950, (**b**) 1100, (**c**) 1150, and (**d**) 1350 m a.s.l. Daily runoff[17] (blue bars, right y-axis) shown for each station group. Light-grey shading shows timing of lake presence. Individual-day shading shows days of hydro-fracture events of lakes within the GNSS array. Velocities are calculated using a sliding least-squares window width of 36 hr, except for 2023/188–196 at the 950s, 2023/195–205 at the 1150s and 1350s, and 2023/193–205 at MHIH, wherein a sliding least-squares window width of 18 hr is used.

Text S1: C1 Cluster Chronology (2022/195)
The C1 hydro-fracture event cluster includes drainages of two of the three instrumented lakes in the 950s (L1A and L1B) and is constrained by Sentinel-2 images on 2022/194, 195, and 196 and a Sentinel-1 image on 2022/194. This cluster comprises five hydro-fracture events occurring on 2022/195 (Table S1). Within the cluster, idealized subglacial blisters produced by the drainages of L1A and L1B produce overlapping, high-tensile-stress regions at the ice-sheet surface, suggesting that these lakes are spatially close enough—and of large-enough volume—to experience elastic stress coupling as they drain (Fig. S1.1b). This interpretation is supported by GNSS observations (Fig. S1.3), which show abrupt changes in across-flow deformation at SQ11, similar to that observed by station NL08 during sequential L1A and L1B hydro-fracture events observed in 2011 and 2013[1,18]. Vertical displacement at SQ11 shows two distinct periods of uplift, with uplift peaks separated by ~0.15 d (~3.6 hr; Fig. S1.3); this uplift record likely indicates the inflation of a single subglacial blister as the two lakes drain in sequence[1,2]. L1A began a period of rapid discharge (>200 m^3 s^{-1}) immediately following the first period of uplift at SQ11, indicating a hydro-fracture sequence of L1B followed by L1A (Fig. S1.3d).

The three other C1 events—L34, L47, and L72—are too far apart to be viable candidates for elastic stress coupling via overlapping blister-sourced stresses unless blisters propagate. If subglacial blisters produced by C1 events propagate along ~200˚ following regions of high subglacial discharge (Fig. S1.1a), then all five events are within the same flood path; only 24 hr is needed for a flood to propagate from L1A down to L34. Given the 24-hr window between the available images, we cannot rule out the possibility that a flood starting at L1B propagated—and triggered the hydro-fracture initiation of—these three other lakes. By contrast, if subglacial floods follow the direction of ice-sheet flow (274–282˚; Table S1), all five events are unlikely to be within the same flood pathway. The events are oriented in an across-flow direction from one another; thus, the C1 cluster is not an example of inter-lake triggering in the up- or down-ice-flow direction.

Outside of the five C1 events, in the down-ice-flow and down-subglacial-pathway directions there exist seven lakes viable for hydro-fracture on 2022/195. Of these seven lakes, there are two moulin-drainage events: one on 2022/195 ([–23, –22] km) located down-flow from L34, and one on 2022/197 ([–24, –2] km) located down-flow from L72 (Supplementary Movie 1). This latter moulin-drainage event occurs one day following the estimated time of subglacial-flood arrivals. In the up-flow direction, within ~7 km inland of C1 events there exist six lakes viable for hydro-fracture on 2022/195. Of these six viable lakes, there is one moulin drainage ([–4, –16] km) located up-flow from L47 that initiates on 2022/197 (Supplementary Movie 1). Farther inland, there are two moulin drainages on 2022/195 and 2022/197 located ~10–15 km up-flow from L1A. Finally, observed strain-rate transients during the C1 events show no anomalous extensional strain-rate transients farther inland than the 950s-to-tiepoints; anomalous basal-uplift transients are only observed for stations within the 950s (Fig. S1.2). In summary, the C1 cluster includes five hydro-fracture and three moulin-drainage events that are physically plausible examples of inter-lake triggering; these eight events comprise 7% of the 121 lakes within the ROI that are viable for hydro-fracture on 2022/195 (Table S8).

Table S1. The 2022 C1 hydro-fracture cluster including the 950s lakes: L1A and L1B.

Lake ID	Time Window [DOY] (GNSS)	Lake Elevation [m a.s.l.]	Euclidean Position [km]	Lake Volume [km^3]	Ice-flow direction [deg]	Within L1A/B subglacial catchment?
L81 (L1A)	195 (195.2)	946	[2, 0]	0.014	282˚	Yes.
L78 (L1B)	195 (195.1)	949	[0, -2]	0.026	279˚	Yes, down catchment.
L72	195	916	[-4, -10]	0.009	275˚	Yes, down catchment.
L47	195	860	[-10, -16]	0.029	281˚	Yes, down catchment.
L34	195	832	[-17, -23]	0.009	274˚	Yes, down catchment.

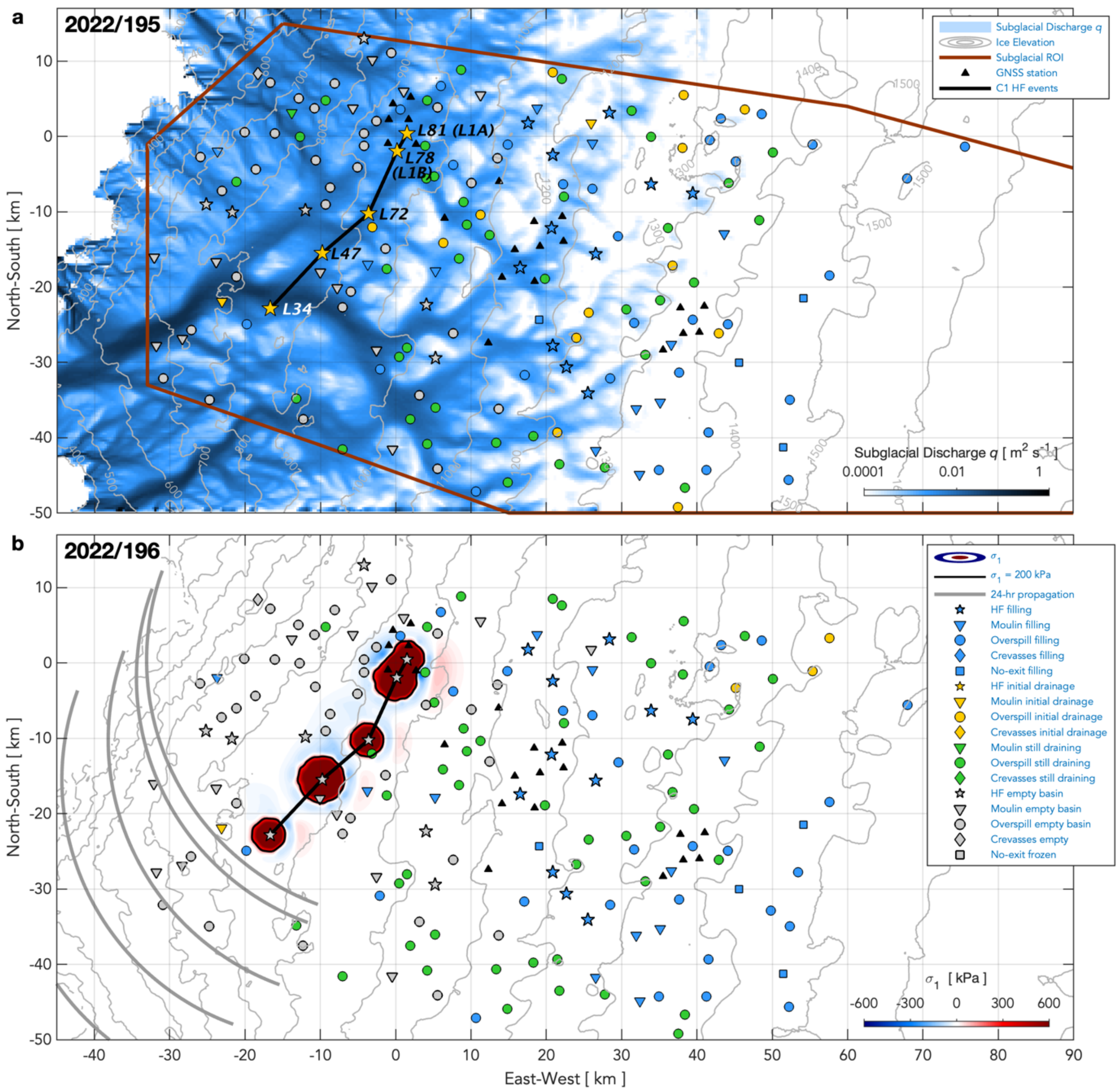

Figure S1.1. Modelled subglacial discharge and ice-sheet surface stress during hydro-fracture event cluster C1 (2022/195). (**a**) Modelled subglacial discharge on 2022/195. Brown line outlines relevant region of interest (ROI) based on subglacial water-flow pathways. Five C1 hydro-fracture events labelled with their identification numbers and linked with black line. Grey contours show ice-sheet surface elevation. (**b**) Modelled maximum principal stress σ_1 from blister opening and basal slip. Black lines show 200-kPa contour in σ_1. Cuspate lines show blister-propagation front 1 d past time of drainage, using propagation aspects of 200–290° from the C1 events. Symbol shapes show lake-drainage mechanism and symbol colors show lake status on (**a**) 2022/195 and (**b**) 2022/196.

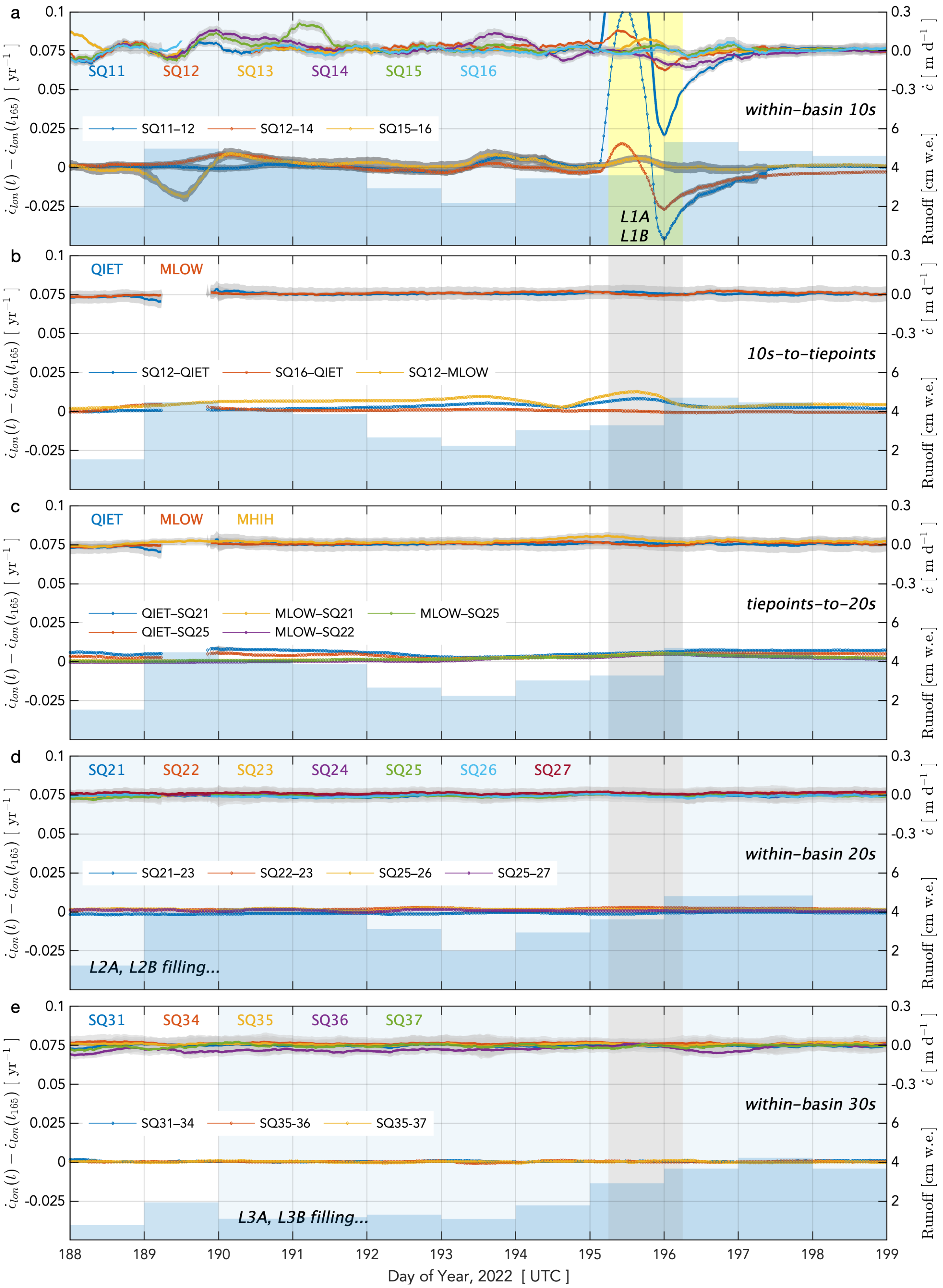

Figure S1.2. Observed strain-rate and basal-uplift transients during cluster C1 (2022/195). (dotted lines, left *y*-axis) Along-flow horizontal strain rates $\dot{\varepsilon}_{lon} \pm 3\sigma$ uncertainty bands (dark grey envelopes) between GNSS-station pairs shown as anomalies relative to $\dot{\varepsilon}_{lon}$ on 2022/165.0 prior to substantial amounts of daily runoff (blue bars, right axis; Noël et al., 2019). (lines, right *y*-axis) Bed separation rate $\dot{c} \pm 3\sigma$ uncertainty bands (light grey envelopes). Panels **a–e** organized by station elevation, moving from lower to higher elevations across the array. Shading shows (light blue) lake presence, (yellow) day of lake drainage(s) within the station cluster, and (grey) day of lake drainages in other regions of the array.

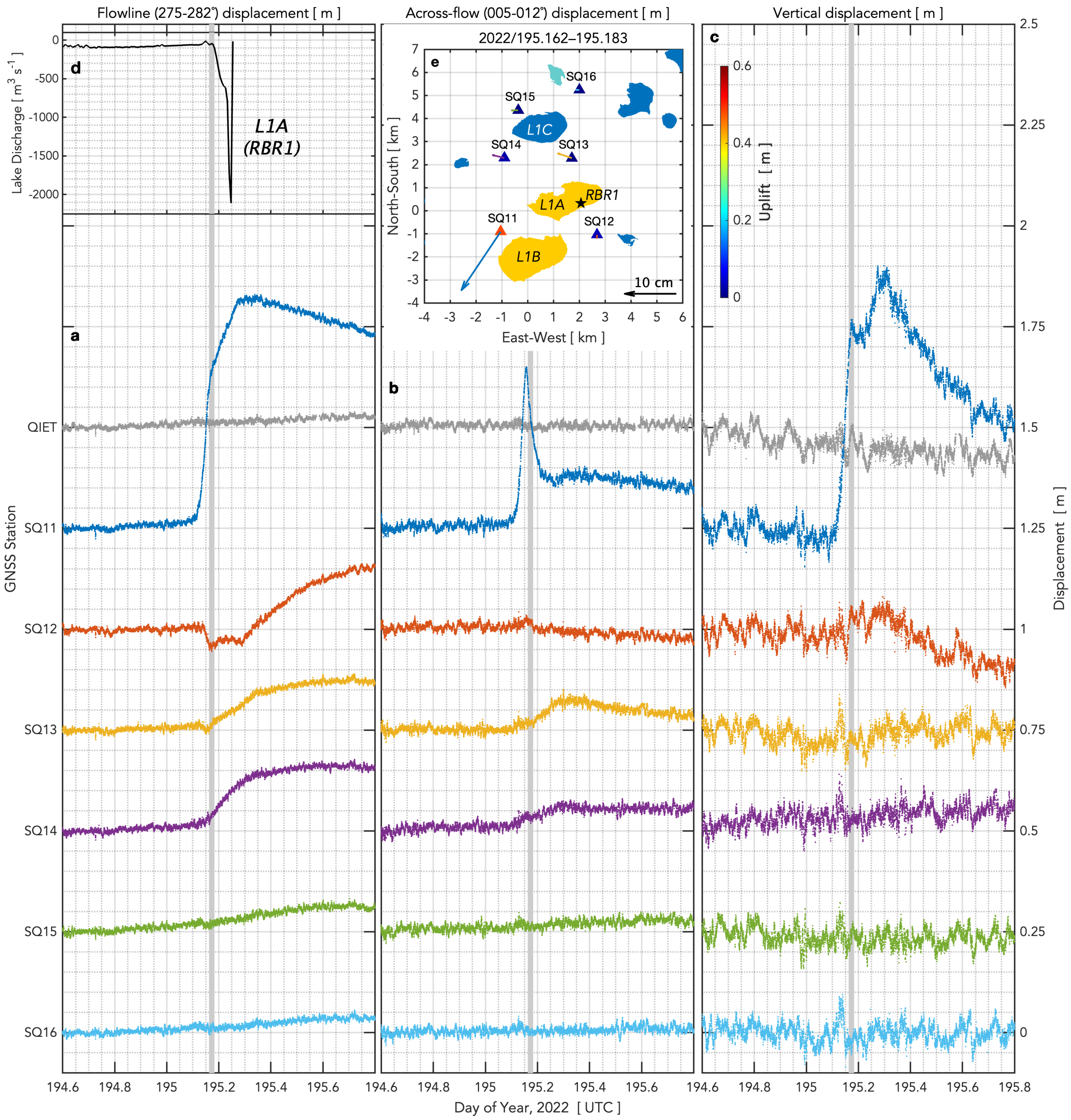

Figure S1.3. Observed ice-sheet horizontal and vertical positions at the 950s and QIET GNSS stations during the C1 cluster (2022/195), which included the hydro-fracture-driven drainages of L1A and L1B. Horizontal displacement accumulated in the (**a**) along- and (**b**) across-flow directions, plotted relative to individual-station flowline direction on 2022/150–160. Positive across-flow displacement shows station displacement to the NNE (005–012˚). (**c**) Vertical displacement. The 15-s resolution horizontal and vertical positions are presented with no temporal smoothing; outliers further than 3σ from the mean value within a moving, centred, 6-hr-width window have been removed. Horizontal and vertical station displacement is detrended based on individual station motion from 2022/194.4–194.8; zeroed to individual-station value on 2022/194.6; and then plotted with 0.25 m of offset between stations. (**d**) Timeseries of L1A lake discharge estimated from RBR1 pressure-logger recordings. (**e**) Station (triangles; colorbar) uplift and (vectors) plan-view displacement for the 0.5-hr time interval given in the panel **e** title and bracketed by grey vertical bars in panels **a–d**. The map orientation and station displacements in panel **e** are not rotated into the along-flow direction. RBR1 location shown with black star. Maximum supraglacial lake margins derived from 2022 FASTER analysis shown for nearby (goldenrod) hydro-fracture, (teal) moulin-, and (dark blue) overspill-type drainages.

Text S2: C2 Cluster Chronology (2022/209–210)

The C2 hydro-fracture event cluster is spatially differentiated into two, three-event subclusters located ~15-km apart (Fig. S2.1). The C2 northern subcluster includes three events occurring during the 96-hr window between Sentinel-2 images on 2022/206 and 210 (Table S2). Within the C2 northern subcluster, idealized subglacial blisters produced by two events (L201, L223) lead to overlapping, high-tensile-stress regions, suggesting that these two lakes experience elastic stress coupling (Fig. S2.1b). The L201 and L223 events could be an example of inter-lake triggering in the inland direction via slippery-patch mechanisms over a ~5.5-km distance (Table S2). The third event (L185) is too far away to be considered a viable candidate for elastic stress coupling unless blisters propagate. If subglacial blisters produced by L201 and/or L223 propagate following regions of high subglacial discharge along ~310˚, then the L185 event is within the same flood pathway; less than 24 h is needed for a flood to propagate from L223 to L185. By contrast, if subglacial floods produced by the L201 and/or L223 events follow the direction of ice-flow (~280–282˚), L185 is unlikely to be within the same flood pathway. Thus, the C2 northern subcluster includes three events that are physically plausible examples of inter-lake triggering.

The C2 southern subcluster includes three lakes located up subglacial-flood pathway from station MHIH; these three events occur within the 48-hr window between Sentinel-2 images on 2022/208 and 210. Idealized subglacial blisters produced by all three events produce overlapping, high-tensile-stress regions, suggesting that these three lakes experience elastic stress coupling. These three events could also be examples of inter-lake triggering in the inland direction via slippery-patch mechanisms over inter-lake spacings of ~3.5–4.5 km. The C2 southern subcluster events all occur within the same flood pathway (Fig. S2.1a). Thus, the C2 southern subcluster includes three hydro-fracture events that are physically plausible examples of inter-lake triggering.

Outside of the C2 events, in the down-flow direction, there exist ~44 lakes viable to hydro-fracture on 2022/210. Of these ~44 lakes, there are no hydro-fracture events from 2022/210–212. There are two moulin-drainage events: one on 2022/210 ([34, –15] km) located down-subglacial-flood path from L223, and one on 2022/211 ([5, –18] km) located down-subglacial-flood path from L2B (Fig. S2.1b). The latter moulin-drainage event commences within the 24-hr subglacial-flood arrivals from the C2 northern and southern subclusters at that location. In the up-flow direction, within ~7 km inland of the C2 events there exist nine lakes viable to hydro-fracture on 2022/210 (Fig. S2.1a). Of these nine lakes, there are no hydro-fracture or moulin-drainage events from 2022/210–212 (Supplementary Movie 1). Farther inland, a moulin drainage of L3B occurs on 2022/210 (Figs. S2.2e), which coincides with moderate along-flow displacement of SQ31, SQ34, and SQ37 (Fig. S2.3). Observed strain-rate transients during the C2 cluster show no anomalous extensional strain-rate transients from 2022/207–211 other than extension observed across the L3B basin on 2022/210 (Fig. S2.2). We interpret the basal-uplift transient at MHIH observed on 2022/209–210 as the subglacial flood from the C2 southern subcluster (Fig. S2.2).

In summary, the C2 northern subcluster includes three hydro-fracture and two moulin-drainage events that are plausible examples of inter-lake triggering; the C2 southern subcluster includes three hydro-fracture events that are plausible examples of inter-lake triggering. A set of 3–5 events comprise 2–4% of the 126 lakes viable for hydro-fracture within the ROI on 2022/210 (Table S8). Considering all six hydro-fracture events of the C2 cluster together as an example of inter-lake triggering of six lakes is not justifiable given the ~15 km, across-flow distance between the northern and southern subclusters.

Table S2: The 2022 C2 hydro-fracture cluster (2022/207–210).

Lake ID	Time Window [DOY]	Lake Elevation [m a.s.l.]	Euclidean Position [km]	Lake Volume [km^3]	Ice-flow direction [deg]	Within same subglacial catchment?
Northern subcluster						
L185	207–210	1213	[28, 3]	0.011	293˚	Yes.
L201	207–210	1286	[34, –6]	0.018	278˚	Yes, up from L185.
L223	207–210	1283	[39, –8]	0.044	281˚	Yes, up from L185.
Southern subcluster						
L168	209–210	1176	[21, –28]	0.007	275˚	Yes.
L173	209–210	1205	[23, –31]	0.005	279˚	Yes, up from L168.
L183	209–210	1232	[25, –34]	0.022	281˚	Yes, up from L168.

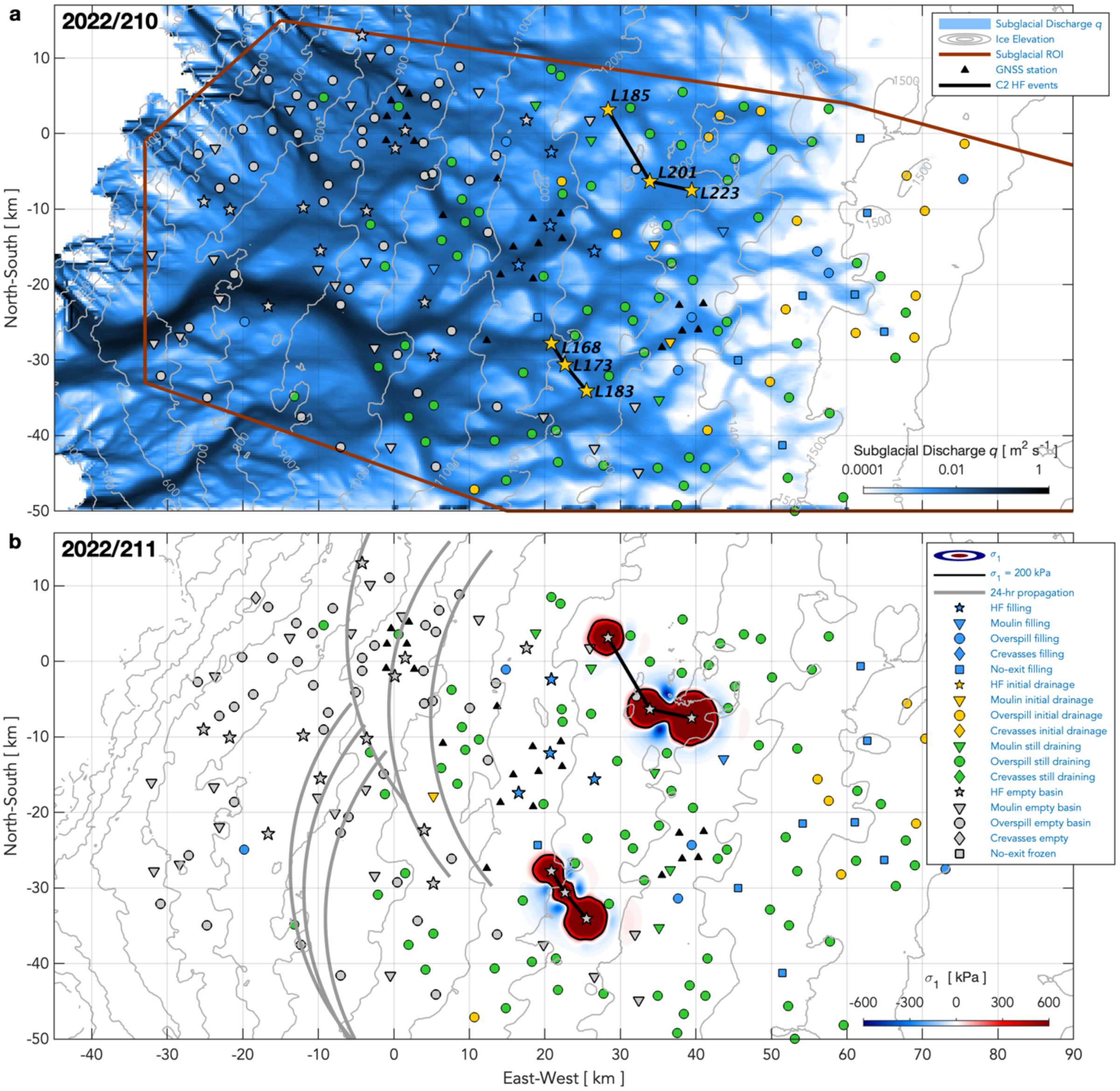

Figure S2.1. Modelled subglacial discharge and ice-sheet surface stress during hydro-fracture event cluster C2 (2022/207–210). (**a**) Subglacial discharge on 2022/210. Six C2 hydro-fracture events labelled with their identification numbers and linked with black lines. (**b**) Modelled maximum principal stress σ_1 from blister opening and basal slip. Black lines show 200-kPa contour in σ_1; red shading shows increase in tensile stress and blue shading shows increased compression. Cuspate lines show blister-propagation front 1 d past time of drainage, using propagation aspects of 240–310˚ from the C2 events. Symbol shapes show lake-drainage mechanism and symbol colors show lake status on (**a**) 2022/210 and (**b**) 2022/211.

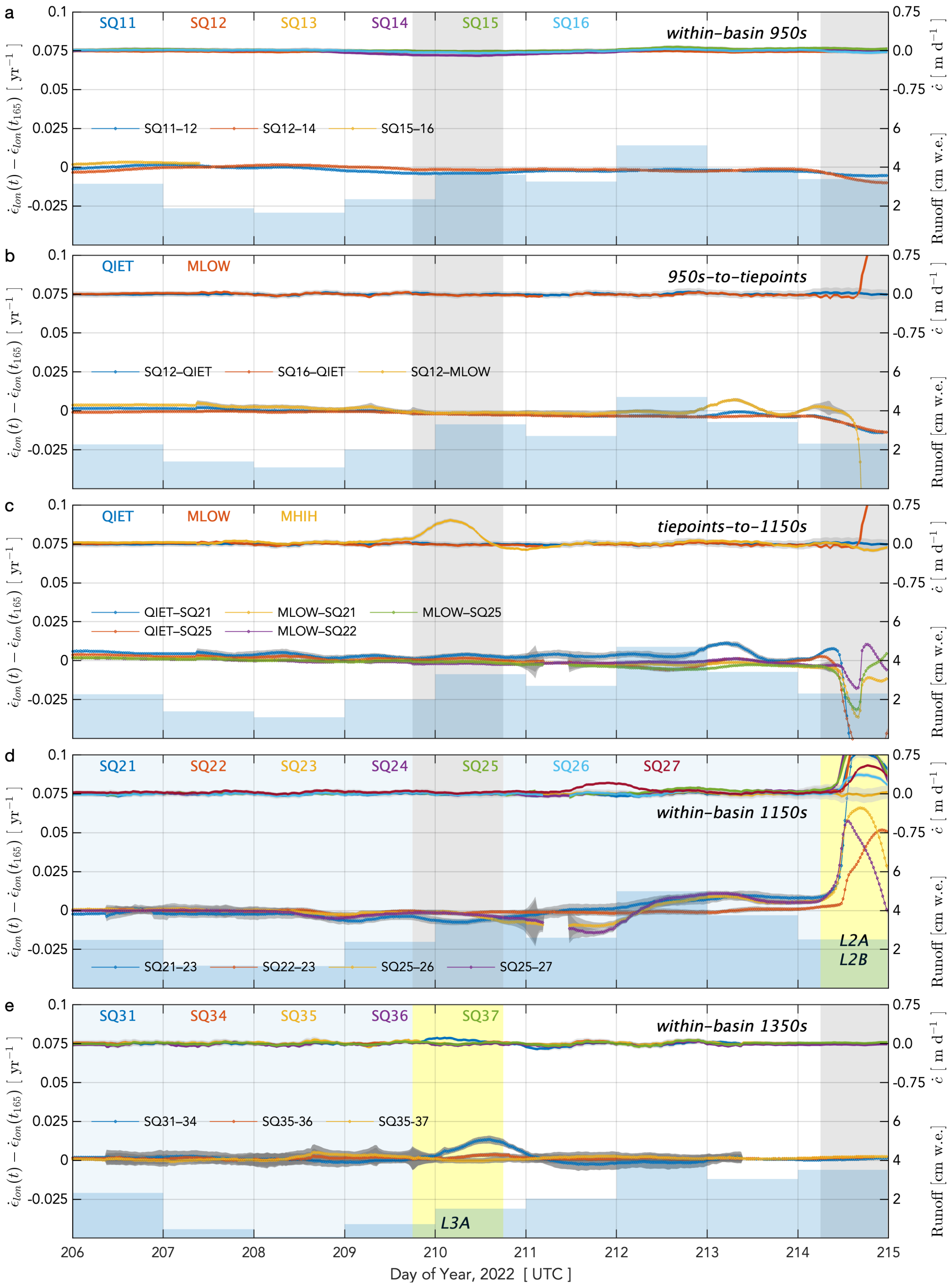

Figure S2.2. Observed strain-rate and basal-uplift transients during cluster C2 (2022/207–210). (dotted lines, left y-axis) Along-flow horizontal strain rates $\dot{\epsilon}_{lon} \pm 3\sigma$ uncertainty bands (dark grey envelopes) between GNSS-station pairs shown as anomalies relative to $\dot{\epsilon}_{lon}$ on 2022/165.0 prior to substantial amounts of daily runoff (blue bars, right axis; Noël et al., 2019). (lines, right y-axis) Bed separation rate $\dot{c} \pm 3\sigma$ uncertainty bands (light grey envelopes). Panels **a–e** organized by station elevation, moving from lower to higher elevations across the array. Shading shows (light blue) lake presence, (yellow) day of lake drainage(s) within the station cluster, and (grey) day of lake drainages in other regions of the array.

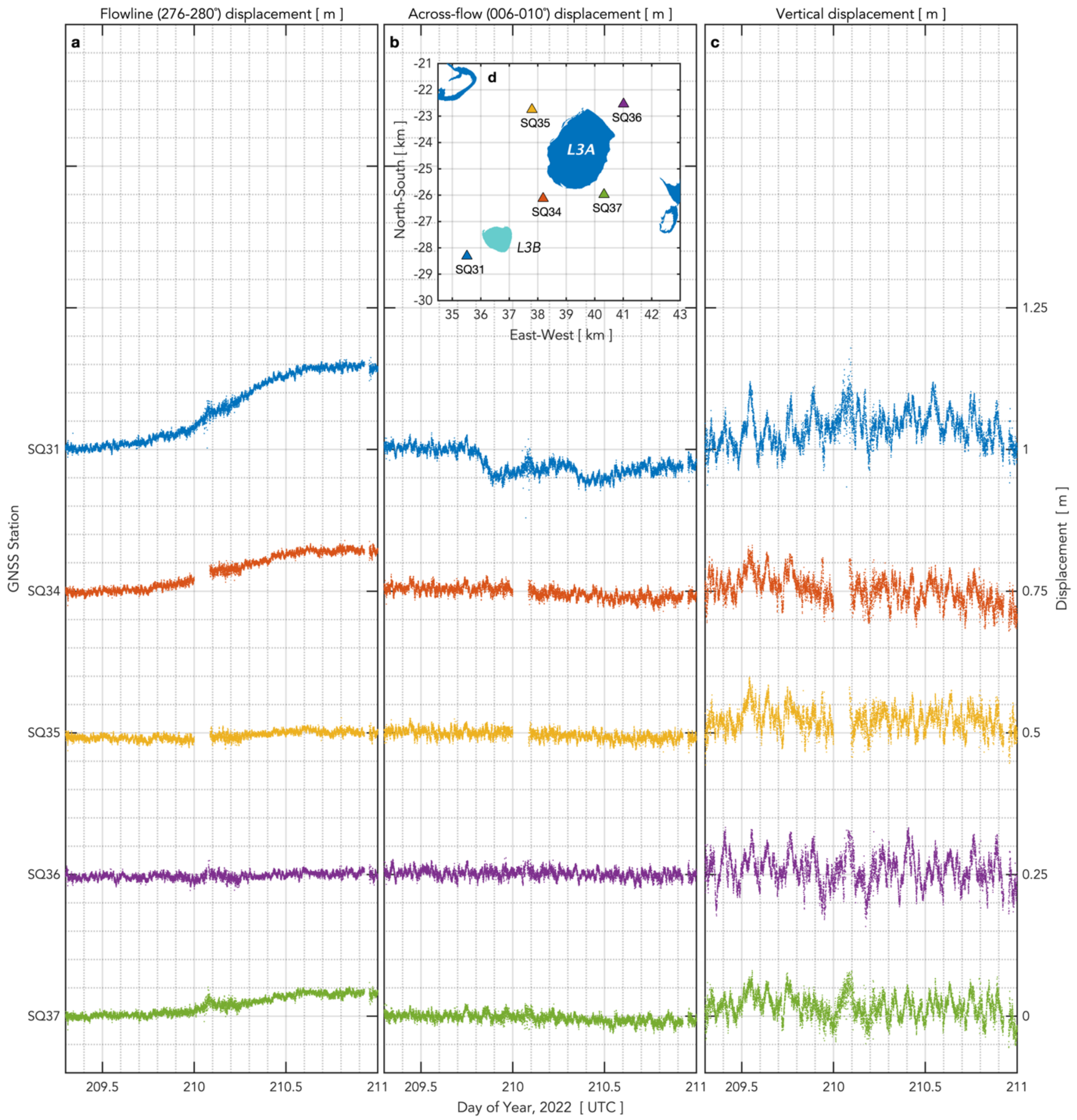

Figure S2.3. Observed ice-sheet horizontal and vertical positions at the 1350s GNSS stations during the C2 hydro-fracture cluster (2022/207–210), which included the moulin-type drainage of L3B. Horizontal displacement accumulated in the (**a**) along- and (**b**) across-flow directions, plotted relative to individual-station flowline direction on 2022/150–160. Positive across-flow displacement shows station displacement to the NNE (006–010˚). (**c**) Vertical displacement. The 15-s resolution horizontal and vertical positions are presented with no temporal smoothing; outliers further than 3σ from the mean value within a moving, centred, 6-hr-width window have been removed. Horizontal and vertical station displacement is detrended based on individual station motion from 2022/209.0–209.5; zeroed to individual-station value on 2022/209.3; and then plotted with 0.25 m of offset between stations. (**d**) (triangles) GNSS stations and maximum supraglacial lake margins derived from 2022 FASTER analysis shown for nearby (teal) moulin- and (dark blue) overspill-type drainages.

Text S3: C3 Cluster Chronology (2022/214–215)

The C3 hydro-fracture event cluster includes drainages of both instrumented lakes in the 1150s (i.e., L2A, L2B) and occurs in the 48-h window between Sentinel-2 images on 2022/213 and 215 (Table S3). Non-instrumented lakes within the C3 cluster include lake L2C located ~5 km up-flow of SQ27 and lake L2D located ~10 km to the north of L2A (Fig. S3.1).

Idealized subglacial blisters produced by the drainages of L2A and L2B create overlapping, high-tensile-stress regions at the ice-sheet surface, suggesting that these lakes experience elastic stress coupling as they drain (Fig. S3.1b). This interpretation is supported by GNSS uplift observations (Fig. S3.3), which track the time of peak uplift from L2A (SQ25: 2022/214.45) to L2B (SQ22: 2022/214.55). We interpret the increase in uplift-peak magnitude from SQ24 (~0.85 m) to SQ22 (~1.00 m) as indicative of the L2B drainage adding water into an existing basal blister initially formed by the L2A drainage. Compared to the L1A and L1B drainages (Fig. S1.3), separate, distinct peaks in uplift are notably missing from GNSS observations of the L2A and L2B drainages (Fig. S3.3). The peak of a third uplift event arrives at SQ27 at 2022/214.63, roughly 2–4 hours after peak uplift of the L2A and L2B events. Taking a flood-propagation speed of 0.4 m s^{-1}, a back-projected peak-uplift time for L2C located 4.6 km inland from SQ27 is 0.13 d prior to 2022/214.63, resulting in an estimated time of peak-uplift at L2C of 2022/214.50. This timing suggests that the drainage of L2C may have been triggered by the drainage of L2A through the slippery-patch mechanism. The lack of a fourth uplift event observed by the 1150s GNSS stations during 2022/213–215—but a speed-up at QIET during this time—suggests that the L2D subglacial flood proceeded in the down-ice-flow (275˚) or down-subglacial-pathway (~280˚) directions, thus avoiding L2A–C. Thus, we hypothesize that the L2A–C hydro-fracture events are plausibly physically related, while the L2D hydro-fracture event is not plausibly related to the L2A–C events (Fig. S3.1b).

Outside of the in-cluster events, in the down-flow direction, there exist ~17 lakes viable to hydro-fracture on 2022/215–217 located along subglacial pathways between C3 events and the western ROI boundary. Of these ~17 lakes, no hydro-fracture or moulin-drainage events occur from 2022/215–217 (Supplementary Movie 1). In the up-flow direction, within ~7 km of the C3 events, there exist five lakes viable for hydro-fracture on 2022/215 (Fig. S3.1a). Of these five viable lakes, there are no hydro-fracture or moulin-drainage events. Farther inland, there is one moulin-drainage event ([43, –14] km) located ~22 km up-flow from L2A on 2022/217 (Supplementary Movie 1). Finally, observed strain-rate transients during the L2A–D hydro-fracture events show no anomalous extensional strain-rate transients across the L3A and L3B basins. In summary, the C3 cluster includes four hydro-fracture events, three of which are plausible examples of inter-lake triggering; these three events comprise 2% of the 126 lakes viable for hydro-fracture within the ROI on 2022/215 (Table S8).

Table S3: The 2022 C3 hydro-fracture lake-drainage cluster including the 1150s lakes: L2A and L2B.

Lake ID	Time Window [DOY] (GNSS)	Lake Elevation [m a.s.l.]	Euclidean Position [km]	Lake Volume [km^3]	Ice-flow direction [deg]	Within same subglacial catchment?
L156 (L2A)	214, 215 (214.45)	1151	[21, –12]	0.062	267˚	Yes.
L143 (L2B)	214, 215 (214.55)	1122	[16, –17]	0.059	269˚	Yes, down from L2A.
L187 (L2C)	214, 215 (214.50)	1213	[27, –16]	0.003	272˚	Yes, up from L2A.
L155 (L2D)	214, 215	1169	[21, –2]	0.055	275˚	Unlikely.

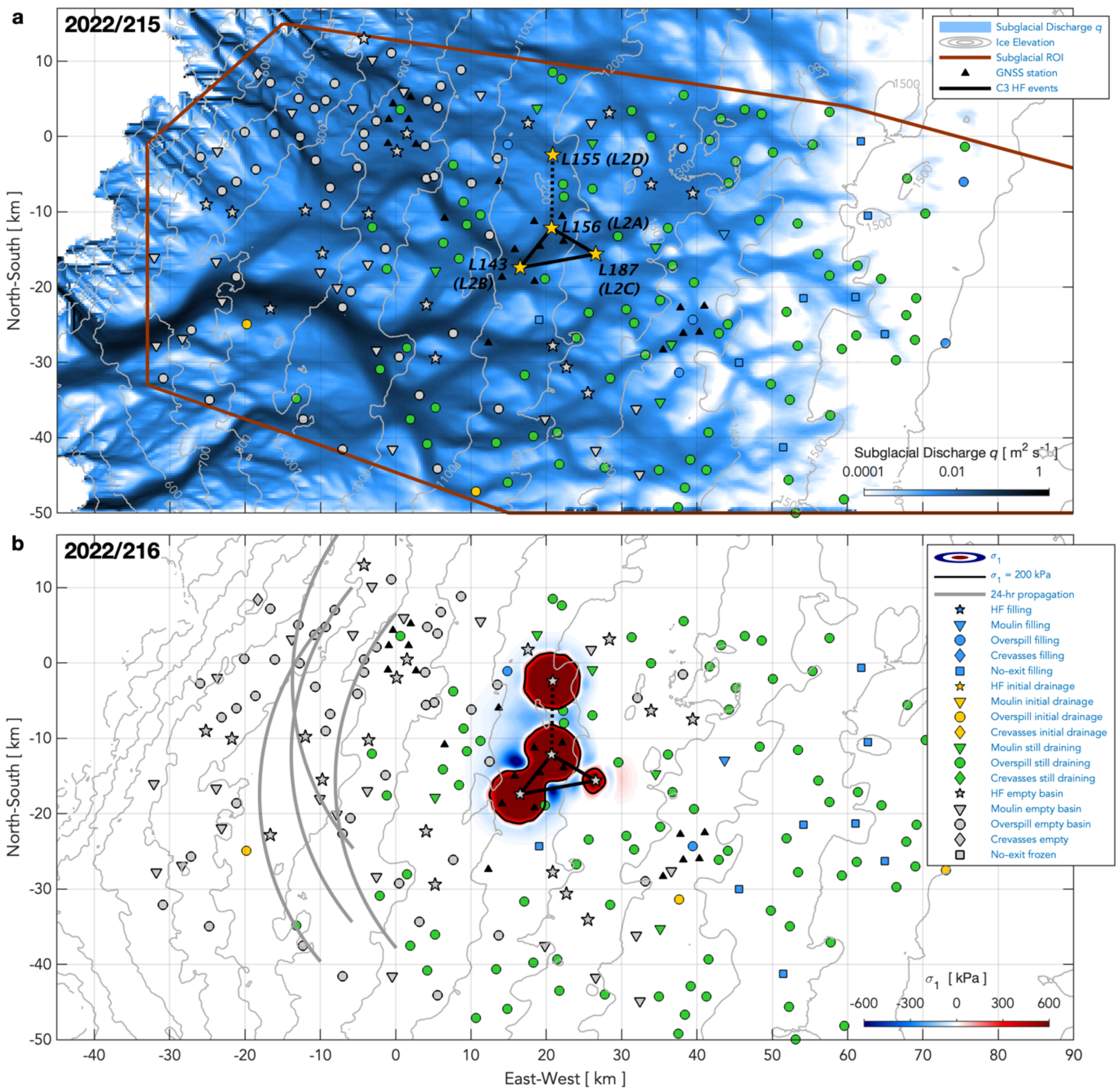

Figure S3.1. Modelled subglacial discharge and ice-sheet surface stress during hydro-fracture event cluster C3 (2022/214–215). (**a**) Subglacial discharge on 2022/214. Four C3 hydro-fracture events labelled with their identification numbers and linked with black lines. (**b**) Modelled maximum principal stress σ_1 from blister opening and basal slip. Black lines show 200-kPa contour in σ_1; red shading shows increase in tensile stress and blue shading shows increased compression. Cuspate lines show blister-propagation front 1 d past time of drainage, using propagation aspects of 230–310˚ from the C3 events. Symbol shapes show lake-drainage mechanism and symbol colors show lake status on (**a**) 2022/214 and (**b**) 2022/215.

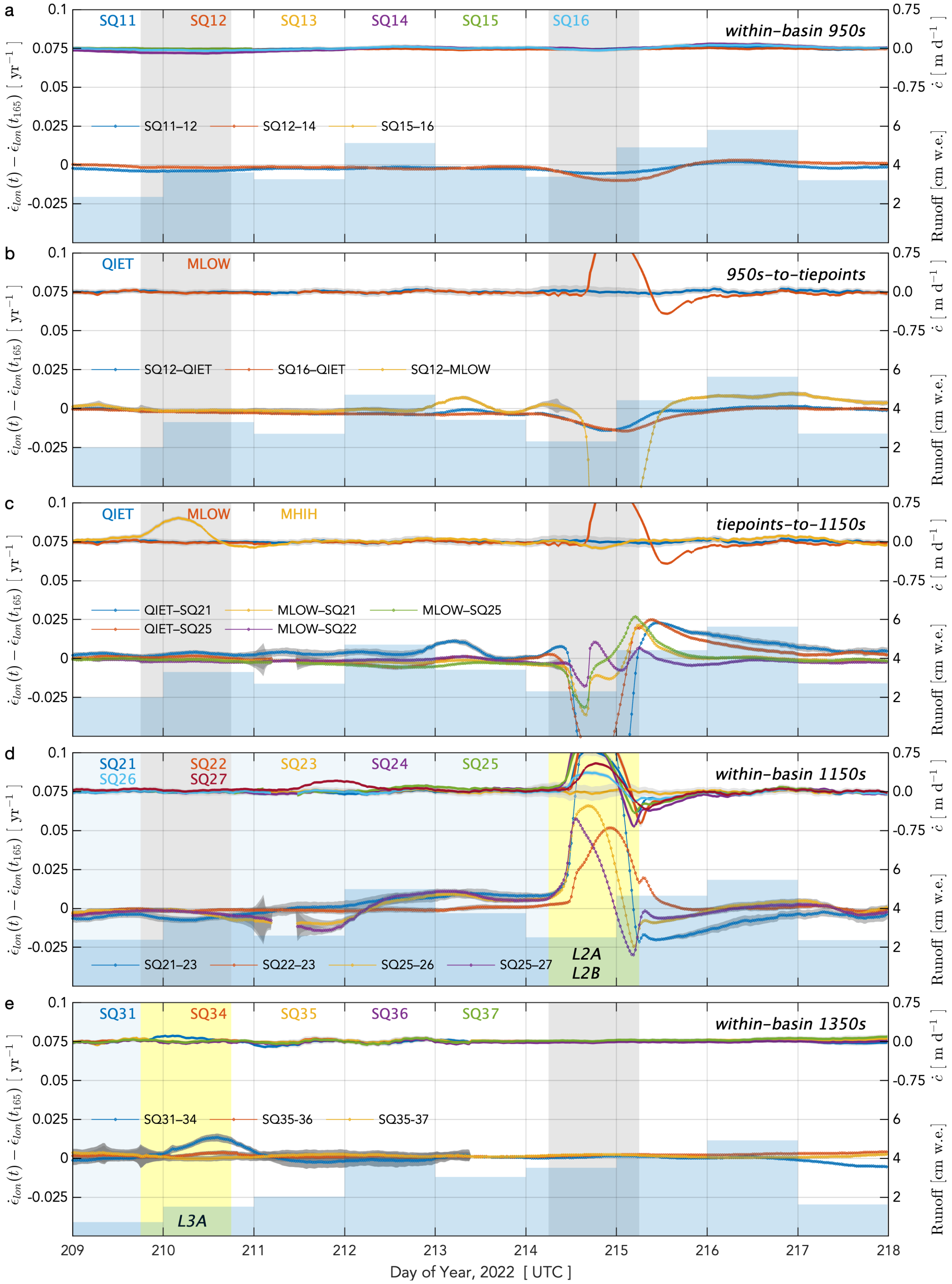

Figure S3.2. Observed strain-rate and basal-uplift transients during cluster C3 (2022/214–215). (dotted lines, left *y*-axis) Along-flow horizontal strain rates $\dot{\varepsilon}_{lon} \pm 3\sigma$ uncertainty bands (dark grey envelopes) between GNSS-station pairs shown as anomalies relative to $\dot{\varepsilon}_{lon}$ on 2022/165.0 prior to substantial amounts of daily runoff (blue bars, right axis; Noël et al., 2019). (lines, right *y*-axis) Bed separation rate $\dot{c} \pm 3\sigma$ uncertainty bands (light grey envelopes). Panels **a–e** organized by station elevation, moving from lower to higher elevations across the array. Shading shows (light blue) lake presence, (yellow) day of lake drainage(s) within the station cluster, and (grey) day of lake drainages in other regions of the array.

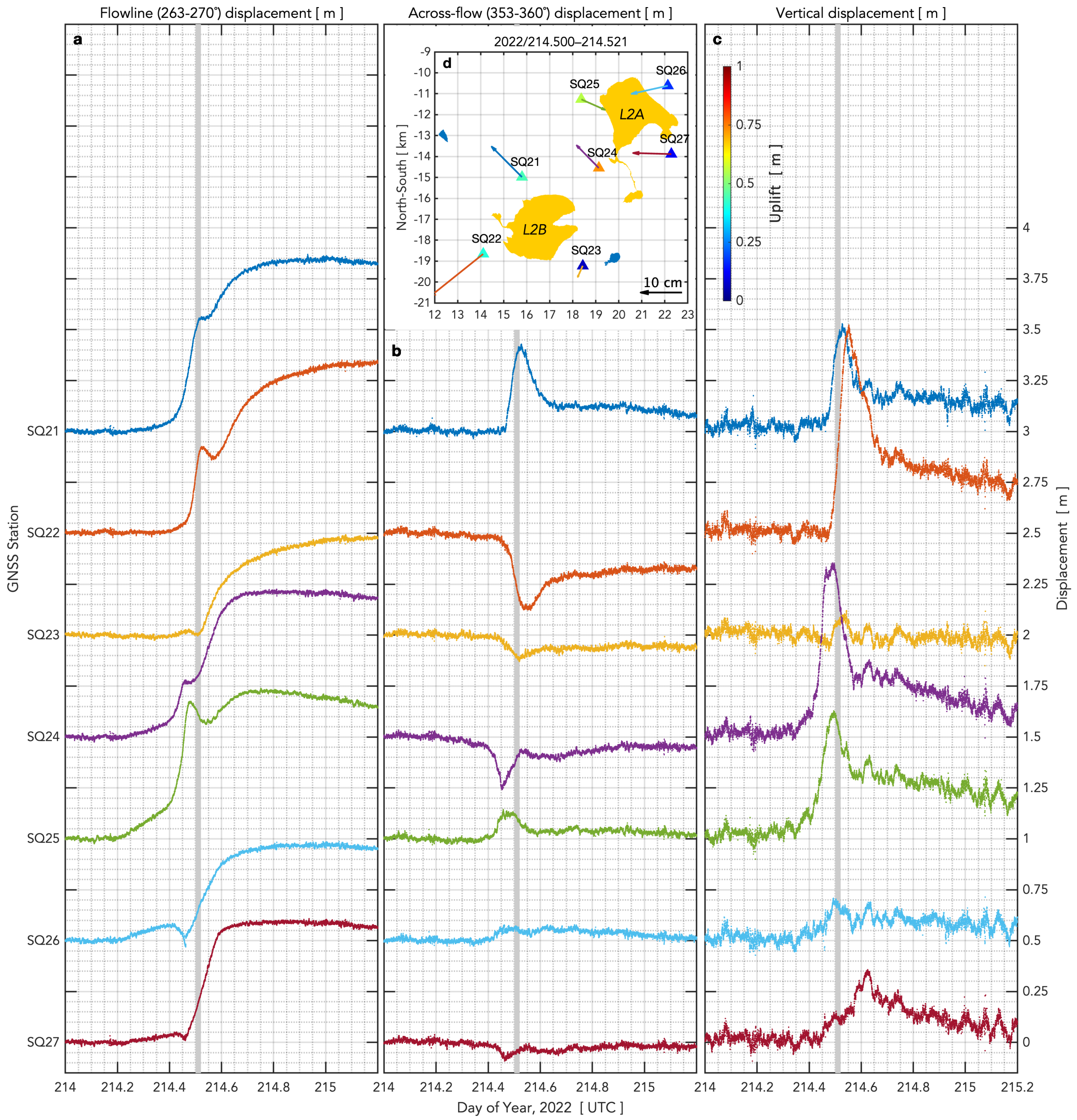

Figure S3.3. Observed ice-sheet horizontal and vertical positions at the 1150s GNSS stations during the C3 hydro-fracture cluster (2022/214–215), which included the hydro-fracture-driven drainages of L2A and L2B. Horizontal displacement accumulated in the (**a**) along- and (**b**) across-flow directions, plotted relative to individual-station flowline direction on 2022/150–160. Positive across-flow displacement shows station displacement to the NNW (353–360˚). (**c**) Vertical displacement. The 15-s resolution horizontal and vertical positions are presented with no temporal smoothing; outliers further than 3σ from the mean value within a moving, centred, 6-hr-width window have been removed. Horizontal and vertical station displacement is detrended based on individual station motion from 2022/213.8–214.1; zeroed to individual-station value on 2022/214.0; and then plotted with 0.5 m of offset between stations. (**d**) Station (triangles; colorbar) uplift and (vectors) plan-view displacement for the 0.5-hr time interval given in the panel **d** title and bracketed by grey vertical bars in panels **a**–**c**. The map orientation and station displacements in panel **d** are not rotated into the along-flow direction. Maximum supraglacial lake margins derived from 2022 FASTER analysis shown for nearby (goldenrod) hydro-fracture and (dark blue) overspill-type drainages.

Text S4: C4 Cluster Chronology (2023/191–193)
The C4 hydro-fracture event cluster includes all three instrumented lakes in the 950s (L1A, L1B, and L1C) and is constrained by Sentinel-2 images on 2023/189, 190, and 193 and a Sentinel-1 image on 2023/189. This cluster includes eight hydro-fracture events occurring on 2023/191–193 as well as the drainage of L1C, which was not identified by the 2023 FASTER analysis (Table S4). Idealized subglacial blisters produced by the L1A and L1B drainages produce overlapping, high-tensile-stress regions, suggesting that these lakes experience elastic stress coupling as they drain (Fig. S4.1b). This interpretation is supported by GNSS observations (Fig. S4.3), which show abrupt changes in across-flow deformation at SQ11, similar to that observed by SQ11 during sequential L1A and L1B hydro-fracture events in 2022 (Fig. S1.3). Vertical displacement at SQ11 shows two distinct periods of uplift, with uplift peaks separated by ~0.05 d (~1.2 hr; Fig. S4.3); this uplift record likely indicates the inflation of a single subglacial blister as L1A and L1B drain in sequence. At stations SQ14 and SQ15, uplift peaks ~0.20 d (~4.8 hr) later in time, likely indicating the end of the L1C drainage.

The remaining six events in the cluster are too far apart to be viable candidates for elastic stress coupling from blister-sourced stresses unless blisters propagate, with the exception of the two, low-volume hydro-fracture events located within ~2 km of each other: L131 and L134. The L75 event could be an example of inter-lake triggering in the inland direction via slippery-patch mechanisms over an ~8-km distance inland from L44. If blisters produced by the eight C4 events propagate following regions of high subglacial discharge along ~200°, then five of the eight events are within the same flood path; only 24 h is needed for a flood to propagate from L1A down to L44 (Fig. S4.1b). By contrast, if subglacial floods follow the direction of ice-sheet flow (~270–290°), C4 events located in up- (L131, L134) and down- (L66) ice-flow directions are likely within the same flood pathway as L1A–C. The L103 event is unlikely to be within the same subglacial catchment as L44 and L75, given the prominent subglacial discharge route to the southwest of L103. Thus, within the cluster, eight of the nine events are plausibly physically related from a combination of stationary and propagating blister-sourced stresses.

Outside of the cluster, in the down-flow direction there exist ~36 lakes viable to hydro-fracture on 2023/193. Within these ~36 lakes, one hydro-fracture event ([–28, –8] km) occurs on 2023/194, which is the estimated day for the subglacial-flood propagation front to reach that location if any C4 events occurred on 2023/193 (Fig. S4.1b). In the up-flow direction, within ~7 km of the C4 events, there exist two viable lakes for hydro-fracture on 2023/193; these two lakes drain via overspill. Farther inland, there are six moulin-drainage events and six hydro-fracture events initiated by 2023/195; these events are considered within the C5 cluster (Text S5). Finally, observed strain-rate transients during the C4 cluster show no anomalous extensional strain-rate transients within the 1150s and 1350s (Fig. S4.2). In summary, the C4 cluster includes nine hydro-fracture events over three days of time; eight events are plausible examples of inter-lake triggering. These eight events—plus an additional, down-ice-flow hydro-fracture event on 2023/194—comprise 6% of the 148 lakes viable for hydro-fracture on 2023/193 (Table S8).

Table S4. The 2023 C4 hydro-fracture cluster including the 950s: L1A, L1B, and L1C. Lake L1C was not identified during by the 2023 FASTER analysis. GNSS-derived drainage timings for L1A–C are given for time of peak uplift recorded by the closest GNSS station to the inferred location of each lakes' subglacial blister.

Lake ID	Time Window [DOY] (GNSS)	Lake Elevation [m a.s.l.]	Euclidean Position [km]	Lake Volume [km^3]	Ice-flow direction [deg]	Within same subglacial catchment?
L44	191,192,193	838	[–12, –10]	0.030	286˚	Yes, down from L1A.
L66	191,192,193	858	[–6, 4]	0.0004	300˚	No, down ice-flow from L1A.
L75	191,192,193	915	[–4, –10]	0.009	277˚	Yes, down from L1A.
L86 (L1A)	191,192,193 (191.20)	943	[2, 1]	0.005	284˚	Yes, down from L1C.
L81 (L1B)	191,192,193 (191.25)	946	[0, –2]	0.015	282˚	Yes, down from L1A.
L1C	(191.45)	--	[0, 4]	--	--	Yes, up from L1A.
L103	191,192,193	1010	[5, –21]	0.0004	269˚	No, in a separate catchment.
L131	191,192,193	1119	[18, 2]	0.0009	285˚	Yes, 16 km up from L1A.
L134	191,192,193	1143	[19, 4]	0.0003	283˚	Yes, 17 km up from L1A.

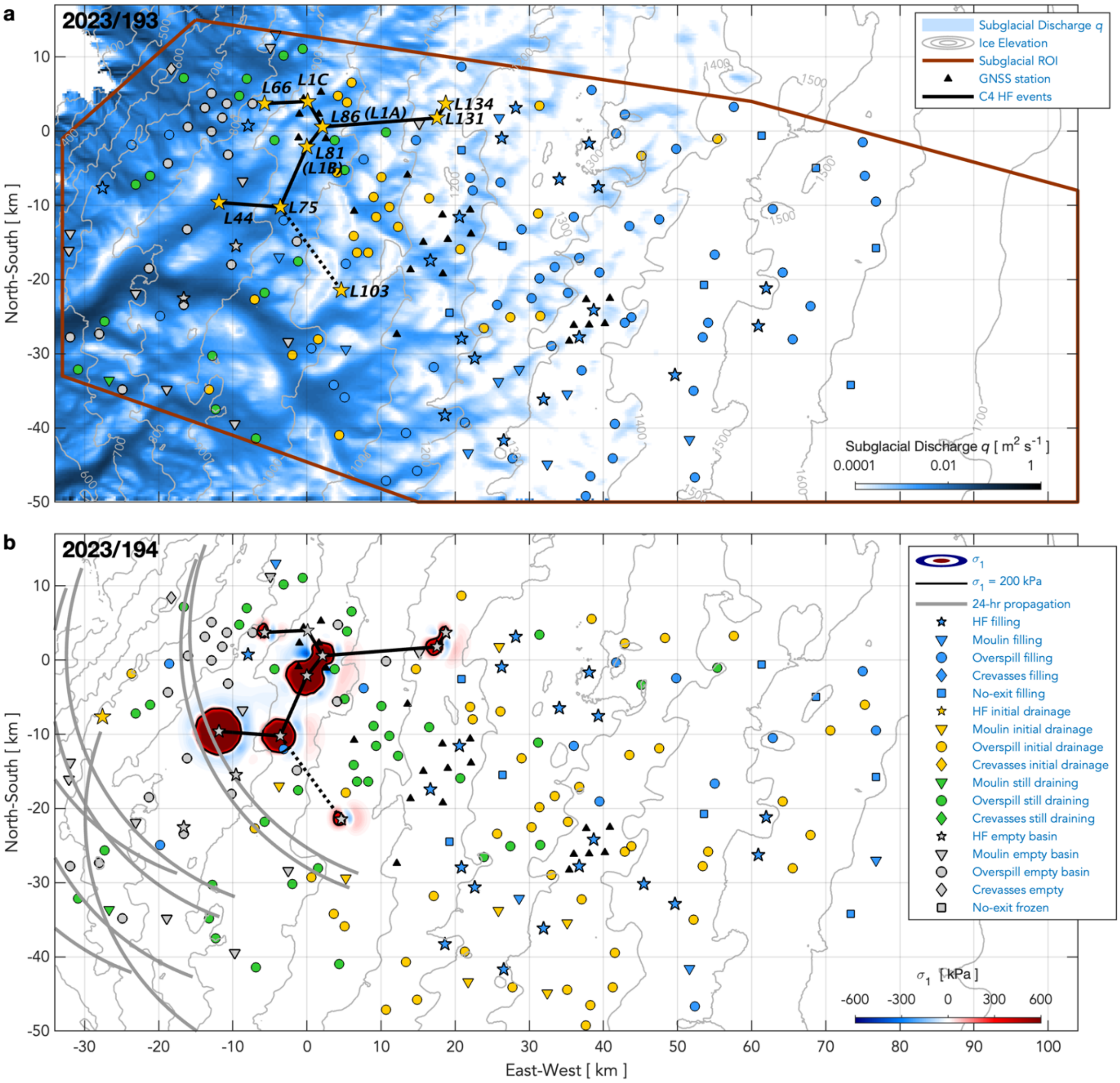

Figure S4.1. Modelled subglacial discharge and ice-sheet surface stress during hydro-fracture event cluster C4 (2023/191–193). (**a**) Subglacial discharge on 2023/193. Nine C4 hydro-fracture events labelled with their identification numbers and linked with black lines. (**b**) Modelled maximum principal stress σ_1 from blister opening and basal slip. Black lines show 200-kPa contour in σ_1; red shading shows increase in tensile stress and blue shading shows increased compression. Cuspate lines show blister-propagation front 1 d past time of drainage, using propagation aspects of 200–290˚ from the C4 events. Symbol shapes show lake-drainage mechanism and symbol colors show lake status on (**a**) 2023/193 and (**b**) 2023/194.

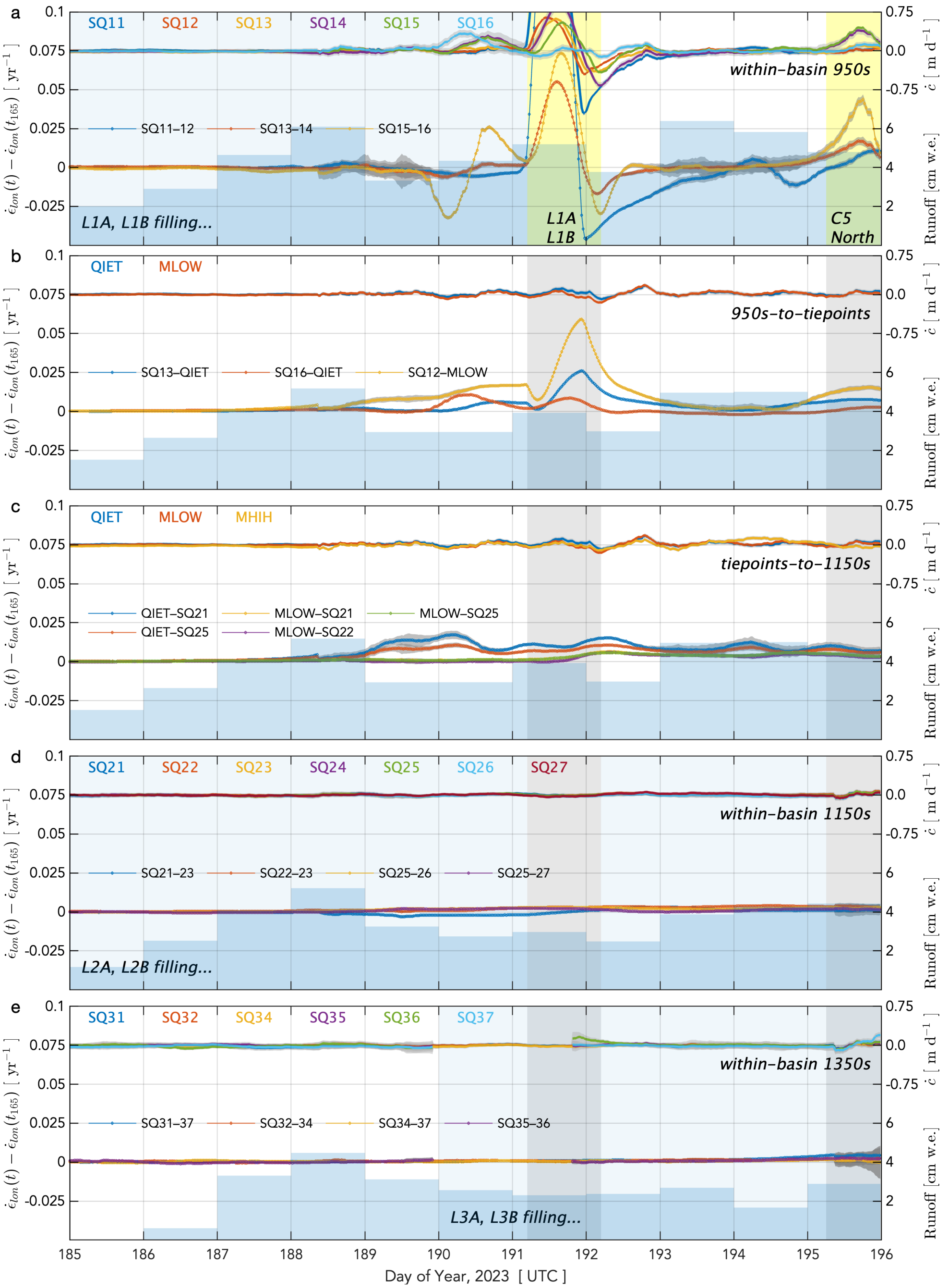

Figure S4.2. Observed strain-rate and basal-uplift transients during cluster C4 (2023/190–193). (dotted lines, left *y*-axis) Along-flow horizontal strain rates $\dot{\varepsilon}_{lon} \pm 3\sigma$ uncertainty bands (dark grey envelopes) between GNSS-station pairs shown as anomalies relative to $\dot{\varepsilon}_{lon}$ on 2023/165.0 prior to substantial amounts of daily runoff (blue bars, right axis; Noël et al., 2019). (lines, right *y*-axis) Bed separation rate $\dot{c} \pm 3\sigma$ uncertainty bands (light grey envelopes). Panels **a–e** organized by station elevation, moving from lower to higher elevations across the array. Shading shows (light blue) lake presence, (yellow) day of lake drainage(s) within the station cluster, and (grey) days of lake drainages in other regions of the array.

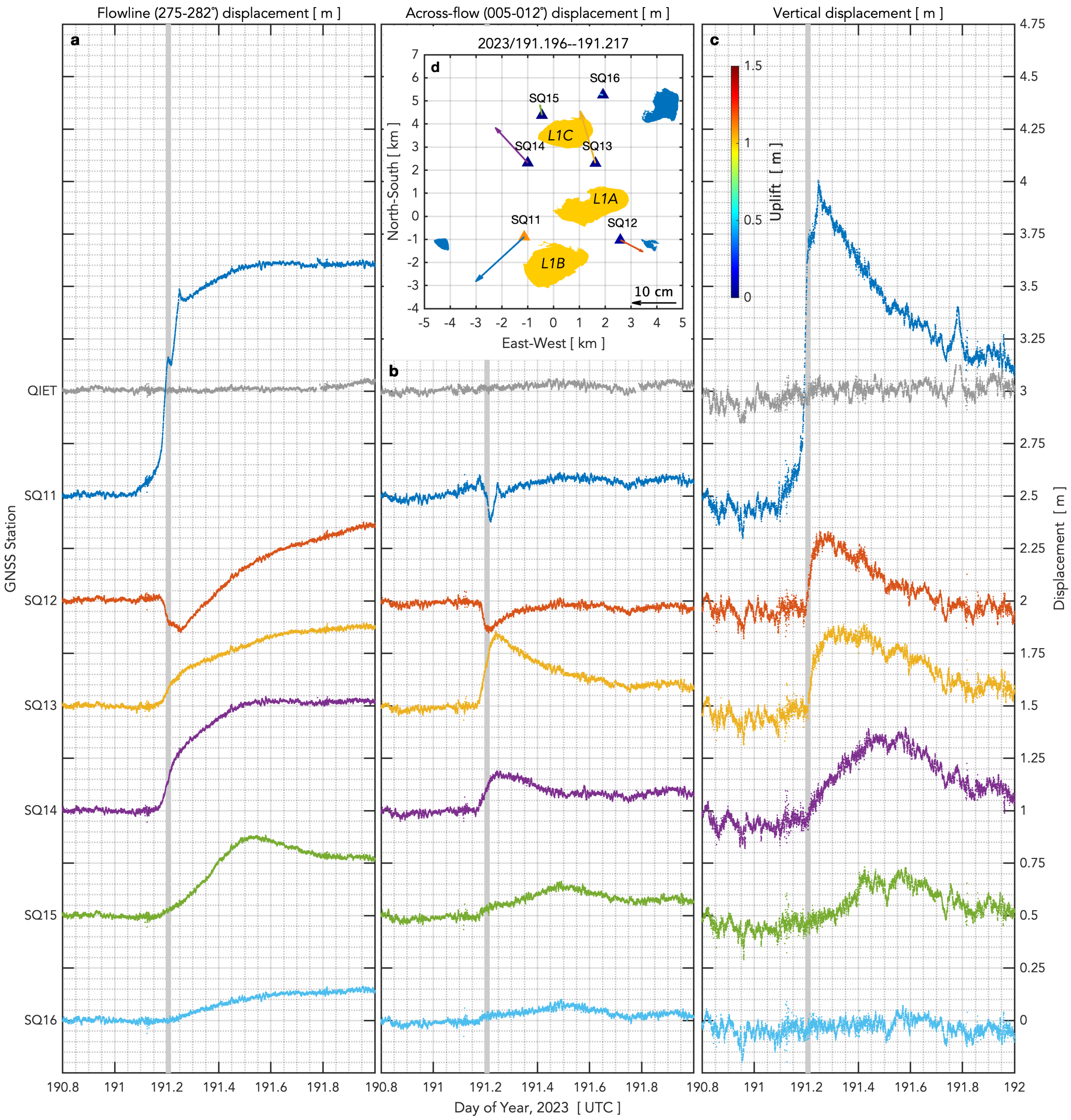

Figure S4.3. Observed ice-sheet horizontal and vertical positions at the 950s and QIET GNSS stations during the C4 hydro-fracture cluster (2023/190–193), which included the hydro-fracture-driven drainages of L1A, L1B, and L1C. Horizontal displacement accumulated in the (**a**) along- and (**b**) across-flow directions, plotted relative to individual-station flowline direction on 2023/150–160. Positive across-flow displacement shows station displacement to the NNE (005–012°). (**c**) Vertical displacement. The 15-s resolution horizontal and vertical positions are presented with no temporal smoothing; outliers further than 3σ from the mean value within a moving, centred, 6-hr-width window have been removed. Horizontal and vertical station displacement is detrended based on individual station motion from 2023/190.6–191.0; zeroed to individual-station value on 2023/190.8; and then plotted with 0.5 m of offset between stations. (**d**) Station (triangles; colorbar) uplift and (vectors) plan-view displacement for the 0.5-hr time interval given in the panel **d** title and bracketed by grey vertical bars in panels **a–c**. The map orientation and station displacements in panel **d** are not rotated into the along-flow direction. Maximum supraglacial lake margins derived from 2023 FASTER analysis shown for nearby (goldenrod) hydro-fracture and (dark blue) overspill-type drainages. The L1C lake margin shown here is the L1C lake margin estimated by the 2022 FASTER analysis.

Text S5: C5 Cluster Chronology (2023/195–201)

The C5 hydro-fracture event cluster is spatially differentiated into two, five-event subclusters located ~15-km apart (Fig. S5.1). This cluster is constrained by Sentinel-2 images on 2023/194, 196, 199, and 200, and a Sentinel-1 image on 2023/201. Considering all ten hydro-fracture events of the C5 cluster together as an example of inter-lake triggering is not justifiable given the many lakes located with the ~15 km across-flow distance between the subclusters that drain via overspill.

The C5 northern subcluster includes five hydro-fracture events occurring within the seven-day window between 2023/194–201 (Table S5). The L154 hydro-fracture event occurs prior to the four other events within this subcluster and is thus removed from analysis of the subcluster. Within the subcluster, idealized subglacial blisters produced by the four events do not produce overlapping, high-tensile-stress regions, suggesting that these lakes are not close enough to experience elastic stress coupling unless subglacial blisters propagate (Fig. S5.1b). However, the L196 event could be an example of inter-lake triggering in the inland direction via slippery-patch mechanisms over a ~5.5-km distance inland of L179 and L194. If subglacial blisters produced by the L179, L194, and/or L196 events propagate following regions of moderate subglacial discharge along ~310˚, then the L162 event is within the same flood pathway; less than 24 h is needed for a flood to propagate from L196 to L162. The L162 event is also likely to be within the L179, L194, and/or L196 flood pathways should these floods follow the direction of ice-flow (~280–300˚). Thus, the C5 northern subcluster includes four hydro-fracture events that are physically plausible examples of inter-lake triggering.

The C5 southern subcluster includes five lakes located up subglacial-flood pathway from GNSS station MHIH; four of the five events occur within the six-day window between Sentinel-2 images on 2023/194 and 200, with the fifth hydro-fracture event constrained by the Sentinel-1 image on 2023/201 (Table S5). Within this subcluster, idealized subglacial blisters produced by two of the five events produce overlapping, high-tensile-stress regions, suggesting that these two lakes (i.e., L150, L152) experience elastic stress coupling as they drain (Fig. S5.1b). The L152 event could be an example of inter-lake triggering in the inland direction via slippery-patch mechanisms over a ~3.5-km distance inland of L150. Considering the modelled subglacial drainage-system pathways estimated for 2023/198, the southern subcluster events fall within two nearby, yet distinct, flood pathways (Fig. S5.1a), with the L150 and L152 events propagating along ~290˚ to beneath MHIH, and the L146, L171, and, potentially, the L181 events propagating along ~270˚ and south of any GNSS stations. Thus, the C5 southern subcluster includes five hydro-fracture events that are physically plausible examples of inter-lake triggering.

Outside of the C5 cluster, in the down-flow direction, there exist ~72 lakes viable to hydro-fracture on 2023/198. Of these ~72 lakes, there are no hydro-fracture events from 2023/198–200 (Fig. S5.1). Of these ~72 lakes, there are four moulin-drainage events: three of these events commence by 2023/198 within and down- subglacial-flood path from the southern subcluster and a fourth commenced on 2023/200 ([–5, 13] km) located down-subglacial-flood path from the northern subcluster. In the up-flow direction, within ~7 km inland of the C5 events there exist twelve lakes viable to hydro-fracture on 2023/198. None of these twelve lakes drain via hydro-fracture in 2023. There is one moulin-drainage event located ~3 km inland of L181 that commences by 2023/198, and one moulin-drainage event located ~7 km up-ice flow from L171 that commences by 2023/200 (Supplementary Movie 2).

Observed strain-rate and basal-uplift transients during the C5 northern subcluster show extensional strain-rate transients within the 950s from 2023/195–196 (Fig. S5.2a), which could be the flood-propagation event ensuant from L154, whose date of drainage is constrained from Sentinel-2 imagery to be 2023/195–196 (Table S5). For the C5 southern subcluster, a basal-uplift transient reaches MHIH on 2023/197 (Fig. S5.2c), which is likely the flood-propagation event ensuant from L150 and L152, whose drainage dates are constrained by Sentinel-1/2 imagery to be from 2023/195–201 (Table

S5). We do not observe additional flood-propagation events in the GNSS observations past 2023/197; however, strain rates within the 1150s and 1350s smoothly diverge from background $\dot{\varepsilon}_{lon}$ from 2023/198 onwards until their eventual drainage via hydro-fracture on 2023/202 and 2023/200, respectively. These events are considered within the C6 hydro-fracture event cluster (Text S6).

In summary, the C5 northern subcluster includes four hydro-fracture events and one moulin-drainage event that are physically plausible examples of inter-lake triggering. The C5 southern subcluster includes five hydro-fracture and five moulin-drainage events that are physically plausible examples of inter-lake triggering. These five (C5 northern) and ten (C5 southern) events respectively comprise 3% and 7% of the 151 lakes viable for hydro-fracture on 2023/198 (Table S8).

Table S5: The 2023 C5 hydro-fracture cluster. The drainage event of the blue-shaded row occurs prior to the rest of the events within the C5 northern subcluster and is thus removed from the C5 northern subcluster.

Lake ID	Time Window [DOY]	Lake Elevation [m a.s.l.]	Euclidean Position [km]	Lake Volume [km^3]	Ice-flow direction [deg]	Within same subglacial catchment?
Northern subcluster						
L154	195–196	1212	[26, –1]	0.008	281°	Yes, down from L179.
L162	197–201	1215	[28, 3]	0.007	296°	Yes, down from L194.
L179	197–199	1290	[34, –7]	0.005	282°	Yes, up from L154.
L194	197–201	1286	[38, –2]	0.002	288°	Yes, up from L162.
L196	197–199	1283	[39, –8]	0.020	282°	Yes, up from L179.
Southern subcluster						
L146	195–200	1190	[19, –38]	0.001	280°	Yes, down from L171.
L150	195–200	1176	[21, –28]	0.006	275°	Yes, down from L152.
L152	195–201	1205	[23, –31]	0.005	281°	Yes, up from L150.
L171	195–200	1287	[27, –42]	0.001	283°	Yes, up from L146.
L181	195–200	1308	[32, –36]	0.005	284°	Yes, up from L171.

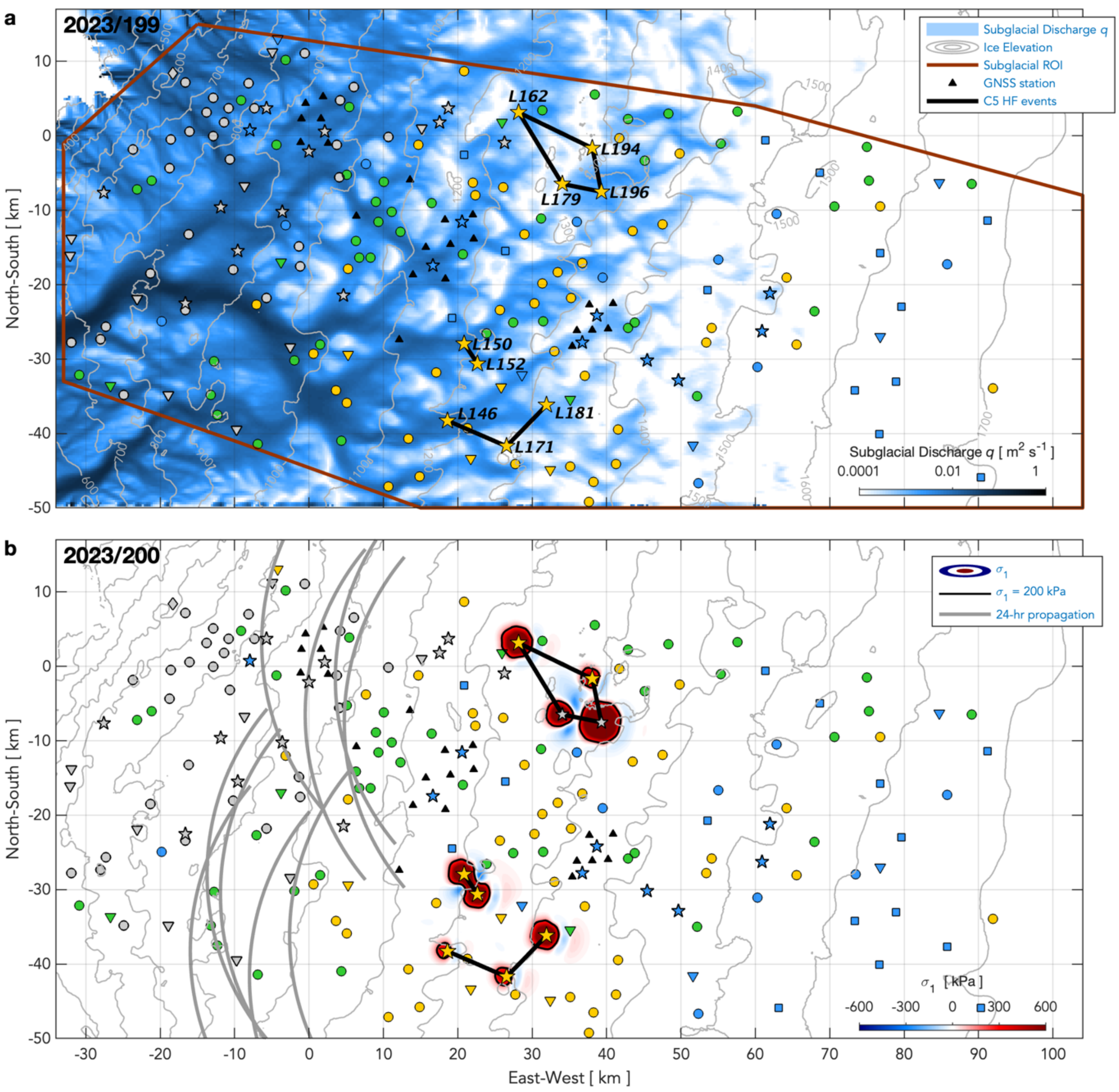

Figure S5.1. Modelled subglacial discharge and ice-sheet surface stress during hydro-fracture event cluster C5 (2023/194–201). (**a**) Subglacial discharge on 2023/199. Nine C5 hydro-fracture events labelled with their identification numbers and linked with black lines. (**b**) Modelled maximum principal stress σ_1 from blister opening and basal slip. Black lines show 200-kPa contour in σ_1; red shading shows increase in tensile stress and blue shading shows increased compression. Cuspate lines show blister-propagation front 1 d past time of drainage, using propagation aspects of 230–310° from the C5 events. Symbol shapes show lake-drainage mechanism and symbol colors show lake status on (**a**) 2023/199 and (**b**) 2023/200; symbol shapes and colors are indicated in the Fig. S4.1 legend.

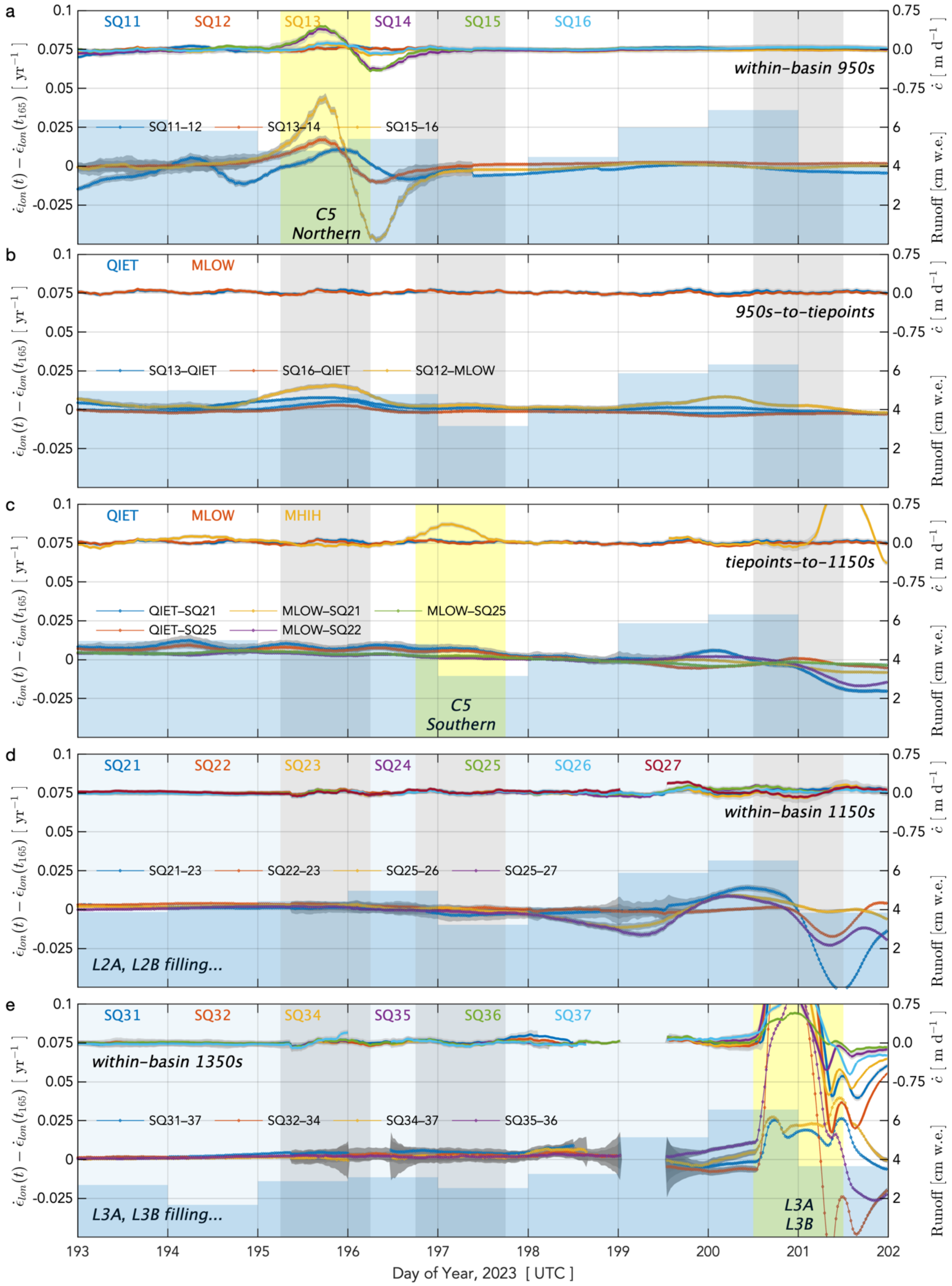

Figure S5.2. Observed strain-rate and basal-uplift transients during cluster C5 (2023/194–201). (dotted lines, left y-axis) Along-flow horizontal strain rates $\dot{\varepsilon}_{lon} \pm 3\sigma$ uncertainty bands (dark grey envelopes) between GNSS-station pairs shown as anomalies relative to $\dot{\varepsilon}_{lon}$ on 2023/165.0 prior to substantial amounts of daily runoff (blue bars, right axis; Noël et al., 2019). (lines, right y-axis) Bed separation rate $\dot{c} \pm 3\sigma$ uncertainty bands (light grey envelopes). Panels **a–e** organized by station elevation, moving from lower to higher elevations across the array. Shading shows (light blue) lake presence, (yellow) day of lake drainage(s) within the station cluster, and (grey) day of lake drainages in other regions of the array.

Text S6: C6 Cluster Chronology (2023/201)

The C6 hydro-fracture event cluster includes drainages of both instrumented lakes in the 1350s (L3A, L3B) and is constrained by a Sentinel-2 image on 2023/200 and a Sentinel-1 image on 2023/201. The cluster includes six events on 2023/201 (Table S6). Within the cluster, idealized subglacial blisters produced by the L3A and L3B events produce overlapping, high-tensile-stress regions, suggesting that these lakes experience elastic stress coupling as they drain (Fig. S6.1). This interpretation is supported by GNSS uplift observations (Fig. S6.3), which track the time of peak uplift from L3A (SQ35: 2023/200.60) to L3B (SQ32: 2023/200.65). We interpret the increase in uplift peak magnitude from SQ34 (~1.45 m) to SQ32 (~1.90 m) as indicative of the L3B drainage adding water into an existing basal blister initially formed by the L3A drainage. In the GNSS observations, a second uplift event peaks first at SQ37 at 2023/200.85, roughly 5–6 hours after the peak uplift of the L3A and L3B drainages. Taking a flood-propagation speed of 0.4 m s^{-1} (Table S9), a back-projected peak-uplift time for L3C located 5.0 km inland from SQ37 is 0.14 d prior to 2023/200.85, resulting in an estimated time of peak-uplift at L3C of 2023/200.71 (Table S6). This drainage timing suggests that the L3C event may have been triggered by slippery-patch stresses produced by the L3A and/or L3B drainages. We do not observe a further uplift peak in the GNSS observations that could plausibly be related to the L3D event, suggesting that floods ensuant from L3C and L3D may have merged by the time of their arrival at SQ37.

The C6 events fall within two nearby, yet distinct, subglacial flood pathways (Fig. S6.1a), with the L3A–D events propagating along ~280˚ to beneath L3A, and then continuing through the drainage system to beneath MHIH. Modelled subglacial drainage-system pathways suggest that the L3E and L3F events would propagate along ~300˚, and thus north of any 1350s GNSS stations. This propagation direction for L3E and L3F is further supported by the absence of departures from background vertical displacements at SQ36, the northernmost station within the 1350s (Fig. S6.3). Thus, within the C6 cluster, four of the six events are plausibly physically related hydro-fracture events.

Outside of the cluster, in the down-flow direction there exist ~36 lakes viable to hydro-fracture on 2023/201–203. Of these ~36 lakes, the L2A and L2B hydro-fracture events commence on 2023/202.75 and 2023/202.85, respectively. Drainage timing for L2A and L2B is taken from the independent observation of lake discharge for the two hydro-fracture events, which shows L2A beginning a period of rapid discharge (>200 m^3 s^{-1}) on 2023/202.75, followed in time roughly 2.4 hr later by L2B beginning a period of rapid discharge on 2023/202.85 (Fig. S6.4d). The drainage timings of L2A and L2B are roughly 24 hr after the L3A–D propagation fronts are estimated to reach the 1150s. Moreover, GNSS-derived bed separation rates within this time window show the two uplift peaks produced by the L3A–D drainages propagating beneath MHIH, not the 1150s; longitudinal strain rates across the 1150s at this time are fluctuating and largely negative (Fig. S6.2). Taken together, the observations suggest that the L2A and L2B hydro-fracture events do not occur because of propagating blisters from the L3A–D drainages traveling directly beneath the L2A and L2B basins.

In the up-flow direction, five lakes viable for hydro-fracture on 2023/201–202 are located within ~7 km of the C6 events; these five lakes drain via overspill (Fig. S6.1). Farther inland, there is one moulin-drainage event ([76, –27] km) located ~15 km inland of L3E that commences on the same day as the C6 events (Figs. S6.1a). In summary, the C6 cluster includes six hydro-fracture events, of which four are plausible examples of inter-lake triggering; these four events comprise 3% of the 157 lakes viable for hydro-fracture on 2023/201 (Table S8).

Table S6: The 2023 C6 hydro-fracture cluster including the 1350s: L3A and L3B. Two rows shaded in green detail the GNSS-constrained L2A and L2B hydro-fracture events that occur after the C6 events. GNSS-derived drainage timings for L3A–C are given for time of peak uplift recorded by the closest GNSS station to the inferred location of each lakes' subglacial blister. Pressure-logger-derived drainage timings for L2A and L2B are given for time of beginning a period of rapid discharge (>200 $m^3 s^{-1}$).

Lake ID	Time Window [DOY] (GNSS)	Lake Elevation [m a.s.l.]	Euclidean Position [km]	Lake Volume [km^3]	Ice-flow direction [deg]	Within same subglacial catchment?
C6 hydro-fracture event cluster						
L3A (208)	201 (200.60)	1354	[39, –24]	0.041*	277˚	Yes.
L3B (201)	201 (200.65)	1315	[37, –28]	0.002	280˚	Yes, down from L3A.
L3C (228)	201 (200.71)	1406	[45, –30]	0.001	281˚	Yes, up from L3A/B.
L3D (238)	201	1443	[50, –33]	0.004	281˚	Yes, up from L3C.
L3E (273)	201	1520	[61, –26]	0.001	287˚	No, north of L3A–D.
L3F (275)	201	1470	[62, –21]	0.002	287˚	No, north of L3A–D.
Characteristics of the 2023 L2A and L2B hydro-fracture events						
L2A (142)	202–206 (202.75)	1153	[21, –12]	0.015	270˚	Up from L2B.
L2B (132)	202–206 (202.80)	1123	[17, –17]	0.041	271˚	Down from L2A.

*Due to the presence of a lake-ice lid that prevents an accurate FASTER-calculated lake surface area for L3A in 2023, the 2023 L3A lake volume is estimated using the 2022 L3A lake surface area.

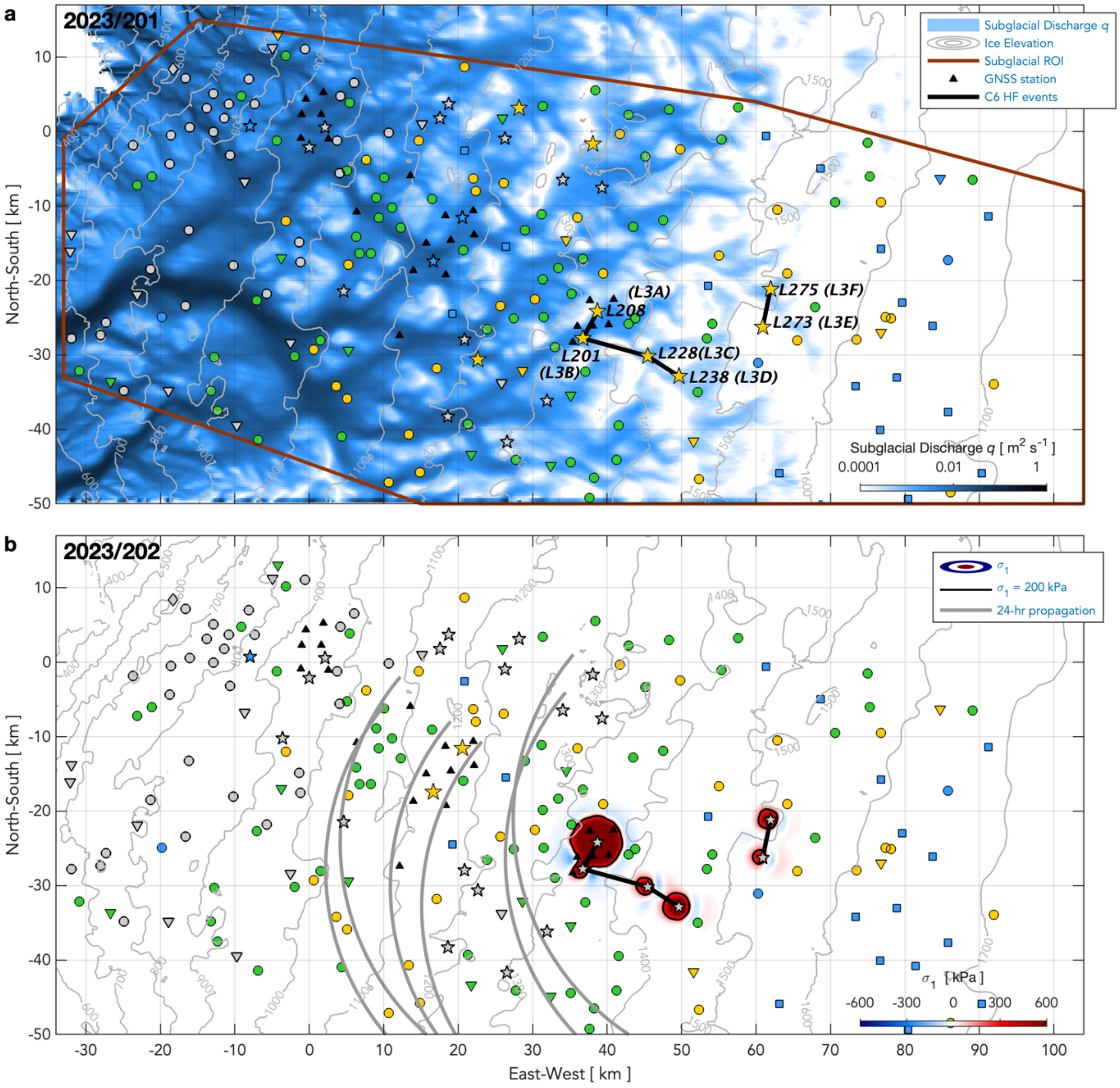

Figure S6.1. Modelled subglacial discharge and ice-sheet surface stress during hydro-fracture event cluster C6 (2023/201). (**a**) Subglacial discharge on 2023/201. Six C6 hydro-fracture events labelled with their identification numbers and linked with black lines. (**b**) Modelled maximum principal stress σ_1 from blister opening and basal slip. Black lines show 200-kPa contour in σ_1; red shading shows increase in tensile stress and blue shading shows increased compression. Cuspate lines show blister-propagation front 1 d past time of drainage, using propagation aspects of 250–310˚ from the C6 events. Symbol shapes show lake-drainage mechanism and symbol colors show lake status on (**a**) 2023/201 and (**b**) 2023/202; symbol shapes and colors are indicated in the Fig. S4.1 legend.

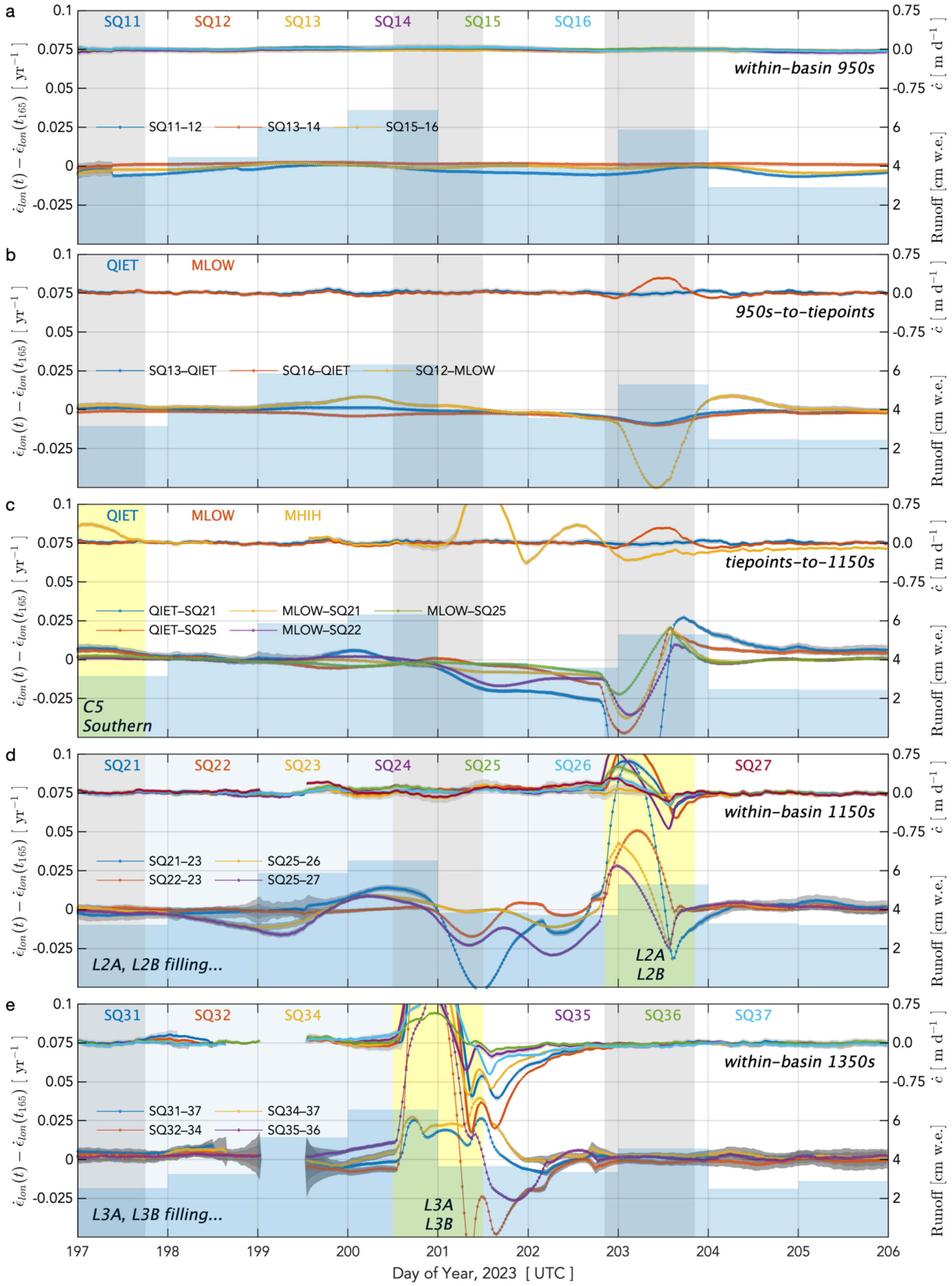

Figure S6.2. Observed strain-rate and basal-uplift transients during cluster C6 (2023/201). (dotted lines, left *y*-axis) Along-flow horizontal strain rates $\dot{\varepsilon}_{lon} \pm 3\sigma$ uncertainty bands (dark grey envelopes) between GNSS-station pairs shown as anomalies relative to $\dot{\varepsilon}_{lon}$ on 2023/165.0 prior to substantial amounts of daily runoff (blue bars, right axis; Noël et al., 2019). (lines, right *y*-axis) Bed separation rate $\dot{c} \pm 3\sigma$ uncertainty bands (light grey envelopes). Panels **a–e** organized by station elevation, moving from lower to higher elevations across the array. Shading shows (light blue) lake presence, (yellow) the day of lake drainage(s) within station cluster, and (grey) days of lake drainages in other regions of the array.

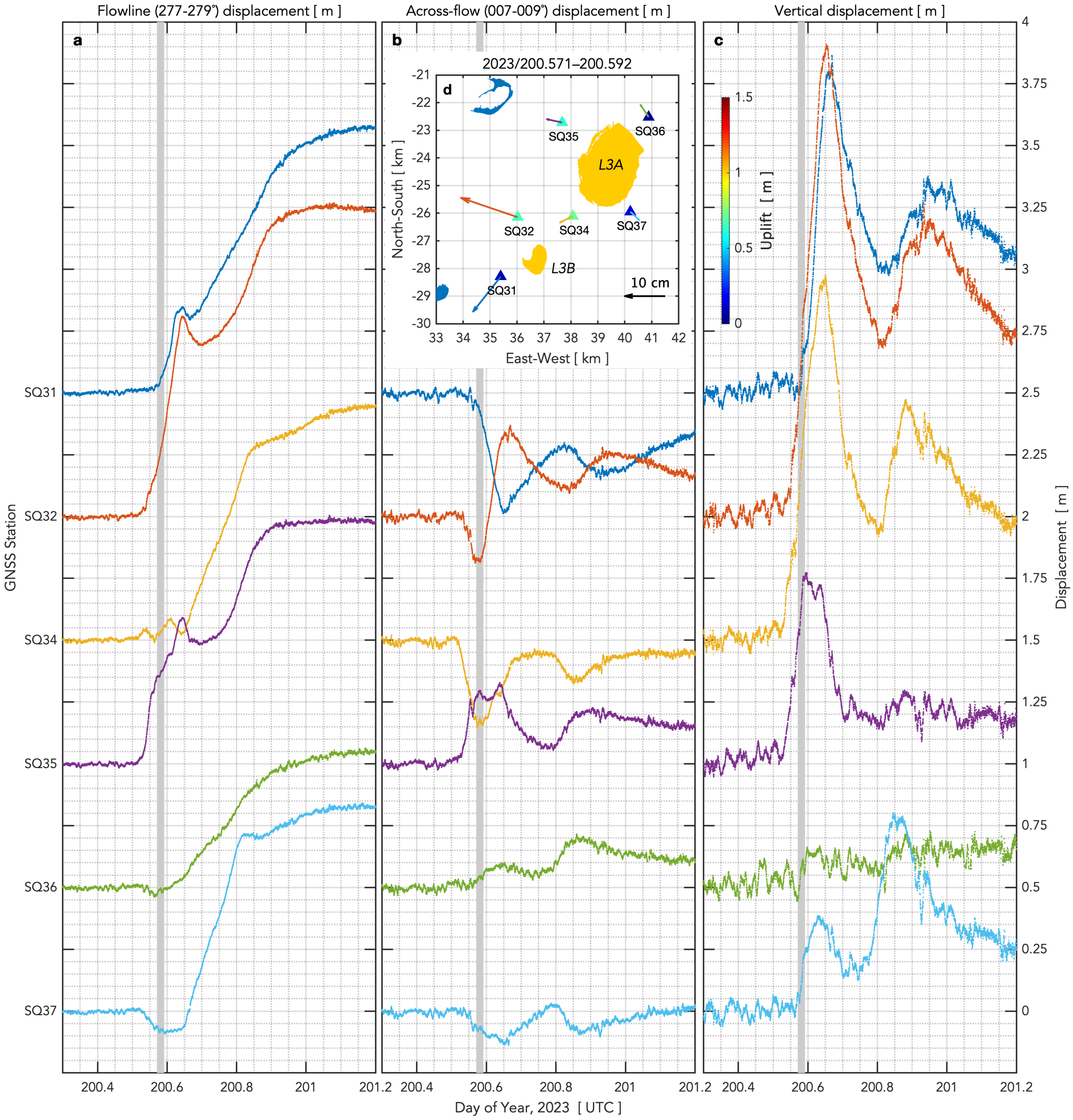

Figure S6.3. Observed ice-sheet horizontal and vertical positions at 1350s GNSS stations during the C6 hydro-fracture cluster (2023/201), which included the hydro-fracture-driven drainages of L3A and L3B. Horizontal displacement accumulated in the (**a**) along- and (**b**) across-flow directions, plotted relative to individual-station flowline direction on 2023/150–160. Positive across-flow displacement shows station displacement to the NNE (007–009˚). (**c**) Vertical displacement. The 15-s resolution horizontal and vertical positions are presented with no temporal smoothing; outliers further than 3σ from the mean value within a moving, centred, 6-hr-width window have been removed. Horizontal and vertical station displacement is detrended based on individual station motion from 2023/200.0–200.4; zeroed to individual-station value on 2023/200.3; and then plotted with 0.5 m of offset between stations. (**d**) Station (triangles; colorbar) uplift and (vectors) plan-view displacement for the 0.5-hr time interval given in the panel **d** title and bracketed by grey vertical bars in panels **a**–**c**. The map orientation and station displacements in panel **d** are not rotated into the along-flow direction. Maximum supraglacial lake margins derived from 2023 FASTER analysis shown for nearby (goldenrod) hydro-fracture and (dark blue) overspill-type drainages. The L3A surface area shown here is the L3A surface area estimated by the 2022 FASTER analysis.

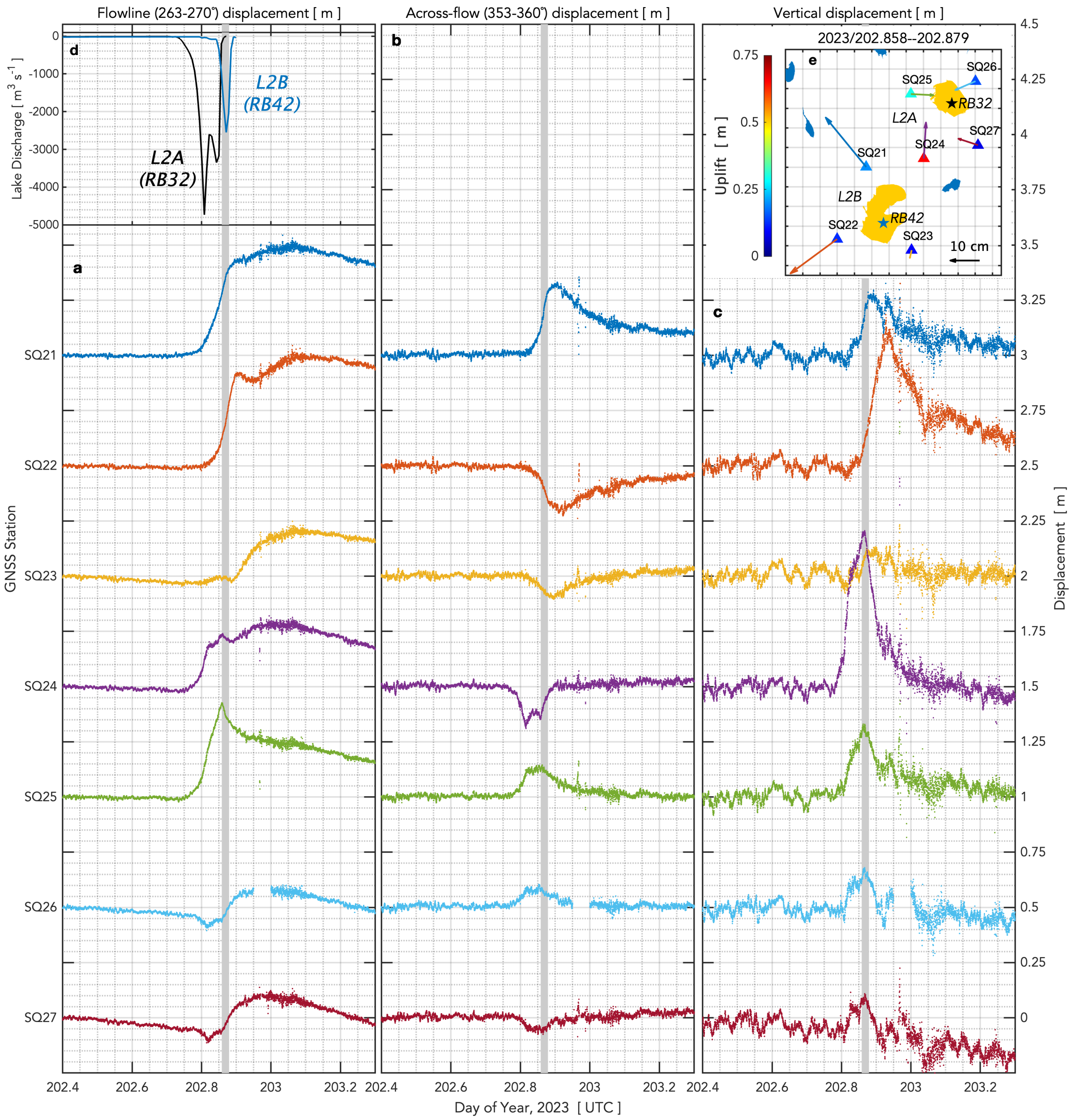

Figure S6.4. Observed ice-sheet horizontal and vertical positions at 1150s GNSS stations during the hydro-fracture-driven drainages of L2A and L2B. Horizontal displacement accumulated in the (**a**) along- and (**b**) across-flow directions, plotted relative to individual-station flowline direction on 2023/150–160. Positive across-flow displacement shows station displacement to the NNW (353–360°). (**c**) Vertical displacement. The 15-s resolution horizontal and vertical positions are presented with no temporal smoothing; outliers further than 3σ from the mean value within a moving, centred, 6-hr-width window have been removed. Horizontal and vertical station displacement is detrended based on individual station motion from 2023/202.2–202.5; zeroed to individual-station value on 2023/202.4; and then plotted with 0.5 m of offset between stations. (**d**) Timeseries of L2A and L2B lake discharge estimated from, respectively, RB32 and RB42 pressure-logger recordings. (**e**) Station (triangles; colorbar) uplift and (vectors) plan-view displacement for the 0.5-hr time interval given in the panel **e** title and bracketed by grey vertical bars in panels **a–d**. The map orientation and station displacements in panel **e** are not rotated into the along-flow direction. Pressure-logger locations shown with stars. Maximum supraglacial lake margins derived from 2023 FASTER analysis shown for nearby (goldenrod) hydro-fracture and (dark blue) overspill-type drainages.

Supplementary Tables

Table S7. Summary statistics of mechanistic lake-drainage catalogues. Percentages are given for water bodies that are lakes within the ice-sheet region of interest (ROI; Fig. 1). FASTER-identified features of water-filled crevasses, streams, slush, and those features truncated by the ROI boundary have been removed from the count.

	2022 melt season:		*2023 melt season:*	
Lake-drainage mechanism:	Count	Percentage of Lakes	Count	Percentage of Lakes
Hydro-fracture	22	11.2%	30	14.4%
In-lake Moulin	28	14.2%	23	11.0%
Overspill	136	69.0%	131	62.7%
Overspilling into another lake	*103*	*52.3%*	*86*	*41.1%*
Overspilling into a downstream moulin	*33*	*16.8%*	*45*	*21.5%*
No-exit frozen	11	5.6%	25	12.0%
Total:	197	100%	209	100%

Table S8. Size of physically plausible hydro-fracture (HF) and moulin-drainage event clusters. Temporal, in-cluster hydro-fracture events are the C1–6 hydro-fracture events identified within the initial temporal analysis (Fig. 1). Associated hydro-fracture and moulin-drainage events are events that fall spatially or temporally outside of the C1–6 events identified within the initial temporal analysis.

Cluster ID	Imagery -date window	Temporal, in-cluster HF events	Physically plausible, in-cluster HF events	Associated HF events	Associated moulin-drainage events	Physically plausible event total	Viable lakes in ROI	Plausible percentage
2022 melt season:								
C1	195	5	5	0	3	8	121	7%
C2 North	207–210	3	3	0	2	5	126	4%
C2 South	209–210	3	3	0	0	3	126	2%
C3	214–215	4	3	0	0	3	126	2%
2023 melt season:								
C4	191–193	9	8	1	0	9	148	6%
C5 North	194–201	5	4	0	1	5	151	3%
C5 South	195–201	5	5	0	5	10	151	7%
C6	201	6	4	0	0	4	157	3%

Table S9. Subglacial flood propagation speeds. Three subglacial flood propagation speeds following hydro-fracture events within our GNSS array estimated from time elapsed between maximum uplift at the two most relevant GNSS stations. Inter-station distance calculated as a straight-line distance between GNSS stations. This distance is not augmented by sinuosity of modelled subglacial-drainage pathways between the two stations; flood-propagation speeds should be interpreted as maximum speeds. Error estimates in propagation speed are calculated using ±0.025-d (±0.6-hr) error estimates on all estimated times of maximum uplift.

Flood event	Time of maximum uplift [DOY] (GNSS)		Inter-station distance [km]	Propagation speed [$m\ s^{-1}$]
2022: L2A/L2B→MLOW	214.552 (SQ22)	214.875 (MLOW)	10.93	0.39 ± 0.07
2023: L2A/L2B→MLOW	202.927 (SQ22)	203.300 (MLOW)	10.94	0.34 ± 0.05
2023: L3A/L3B→MHIH	200.654 (SQ32)	201.263 (MHIH)	23.93	0.45 ± 0.04

Table S10. Station-pair choices for estimating vertical strain rate $\dot{\varepsilon}_{zz}$, and estimated 1σ errors in longitudinal strain rates $\delta\dot{\varepsilon}_{lon}$, for each along-flow station pair, averaged over the melt-season window analysed in both years (DOY 165–230).

	2022			2023		
Station	Along-flow station pair	$\overline{\delta\dot{\varepsilon}_{lon}}$ [yr^{-1}]	Across-flow station pair	Along-flow station pair	$\overline{\delta\dot{\varepsilon}_{lon}}$ [yr^{-1}]	Across-flow station pair
SQ11	SQ11–SQ12	1.9×10^{-4}	SQ11–SQ14	SQ11–SQ12	1.9×10^{-4}	SQ11–SQ14
SQ12	SQ12–QIET	7.0×10^{-5}	SQ12–SQ14	SQ12–QIET	8.3×10^{-5}	SQ12–SQ14
SQ13	SQ13–QIET	5.5×10^{-5}	SQ13–SQ16	SQ13–QIET	7.5×10^{-5}	SQ13–SQ16
SQ14	SQ12–SQ14	1.8×10^{-4}	SQ14–SQ15	SQ12–SQ14	1.8×10^{-4}	SQ14–SQ15
SQ15	SQ15–SQ16	3.2×10^{-4}	SQ14–SQ15	SQ15–SQ16	3.2×10^{-4}	SQ14–SQ15
SQ16	SQ16–QIET	6.6×10^{-5}	SQ14–SQ16	SQ16–QIET	7.4×10^{-5}	SQ14–SQ16
SQ21	SQ21–SQ24	3.4×10^{-4}	SQ21–SQ22	SQ21–SQ24	2.5×10^{-4}	SQ21–SQ22
SQ22	SQ22–SQ23	1.8×10^{-4}	SQ22–SQ24	SQ22–SQ23	1.9×10^{-4}	SQ22–SQ24
SQ23	SQ23–SQ31	5.1×10^{-5}	SQ23–SQ24	SQ23–SQ31	5.1×10^{-5}	SQ23–SQ24
SQ24	SQ24–SQ27	2.4×10^{-4}	SQ24–SQ26	SQ24–SQ27	2.5×10^{-4}	SQ24–SQ26
SQ25	SQ25–SQ26	2.2×10^{-4}	SQ25–QIET	SQ25–SQ26	2.2×10^{-4}	SQ25–QIET
SQ26	SQ25–SQ26	2.2×10^{-4}	SQ26–QIET	SQ25–SQ26	2.2×10^{-4}	SQ26–QIET
SQ27	SQ27–SQ35	5.8×10^{-5}	SQ26–SQ27	SQ27–SQ35	5.3×10^{-5}	SQ26–SQ27
SQ31	SQ31–SQ34	3.0×10^{-4}	SQ31–SQ34	SQ31–SQ34	3.6×10^{-4}	SQ31–SQ32
SQ32	No recordings.	--	No recordings.	SQ32–SQ34	4.3×10^{-4}	SQ31–SQ32
SQ34	SQ34–SQ37	3.4×10^{-4}	SQ34–SQ35	SQ34–SQ37	4.0×10^{-4}	SQ34–SQ35
SQ35	SQ35–SQ36	2.3×10^{-4}	SQ35–SQ37	SQ35–SQ36	2.6×10^{-4}	SQ35–SQ37
SQ36	SQ35–SQ36	2.3×10^{-4}	SQ34–SQ36	SQ35–SQ36	2.6×10^{-4}	SQ34–SQ36
SQ37	SQ34–SQ37	3.4×10^{-4}	SQ36–SQ37	SQ34–SQ37	4.0×10^{-4}	SQ36–SQ37
MLOW	MLOW–QIET	1.2×10^{-4}	MLOW–QIET	MLOW–QIET	1.5×10^{-4}	MLOW–QIET
MHIH	MHIH–SQ31	3.1×10^{-5}	MHIH–SQ22	MHIH–SQ31	4.0×10^{-5}	MHIH–SQ22
QIET	QIET–SQ12	7.0×10^{-5}	QIET–SQ16	QIET–SQ12	8.3×10^{-5}	QIET–SQ16

Table S11. Estimated 1σ errors in bed-separation rate $\delta\dot{c}$ for each station averaged over the melt-season window analysed in both years (DOY 165–230). Ice-surface elevations, ice-sheet thicknesses H, and errors in ice-sheet thicknesses δH are from BedMachine v.5[15,16].

				2022	2023
Station	Ice-surface elevation [m a.s.l.]	Ice-sheet thickness, H [m]	δH [m]	$\overline{\delta\dot{c}}$ [m d^{-1}]	$\overline{\delta\dot{c}}$ [m d^{-1}]
SQ11	986	1019	14	0.014	0.014
SQ12	996	971	12	0.008	0.007
SQ13	979	958	18	0.008	0.009
SQ14	968	908	24	0.016	0.016
SQ15	975	726	30	0.017	0.015
SQ16	974	832	17	0.010	0.011
SQ21	1164	1199	37	0.012	0.015
SQ22	1149	1386	30	0.010	0.013
SQ23	1162	1058	36	0.008	0.008
SQ24	1191	1322	23	0.012	0.015
SQ25	1197	1178	14	0.011	0.013
SQ26	1204	1217	31	0.011	0.012
SQ27	1201	1113	21	0.007	0.009
SQ31	1361	1207	58	0.012	0.020
SQ32	1377	1170	26	No recordings.	0.021
SQ34	1379	1137	18	0.013	0.019
SQ35	1389	1153	45	0.009	0.013
SQ36	1398	1125	14	0.009	0.012
SQ37	1388	1115	25	0.012	0.019
MLOW	1059	1194	32	0.005	0.010
MHIH	1137	1100	90	0.003	0.008
QIET	1126	905	24	0.005	0.009

Supplementary Information References

1. Stevens, L. A. *et al.* Elastic Stress Coupling Between Supraglacial Lakes. *J. Geophys. Res. Earth Surf.* **129**, e2023JF007481 (2024).
2. Lai, C.-Y. *et al.* Hydraulic transmissivity inferred from ice-sheet relaxation following Greenland supraglacial lake drainages. *Nat. Commun.* **12**, 3955 (2021).
3. Rines, J., Lai, C.-Y. & Wang, Y. Longitudinal stress induced by basal slippery patch. Preprint at https://doi.org/10.31223/X50J2P (2026).
4. Williamson, A. G., Banwell, A. F., Willis, I. C. & Arnold, N. S. Dual-satellite (Sentinel-2 and Landsat 8) remote sensing of supraglacial lakes in Greenland. *The Cryosphere* **12**, 3045–3065 (2018).
5. Okada, Y. Surface deformation due to shear and tensile faults in a half-space. *Bull. Seismol. Soc. Am.* **75**, 1135–1154 (1985).
6. Krawczynski, M. J., Behn, M. D., Das, S. B. & Joughin, I. Constraints on the lake volume required for hydro-fracture through ice sheets. *Geophys. Res. Lett.* **36**, 2008GL036765 (2009).
7. Hoffman, M. J. *et al.* Widespread Moulin Formation During Supraglacial Lake Drainages in Greenland. *Geophys. Res. Lett.* **45**, 778–788 (2018).
8. Stevens, L. A. *et al.* Tidewater-glacier response to supraglacial lake drainage. *Nat. Commun.* **13**, 6065 (2022).
9. Tobin, S. & Neufeld, J. A. Evolution of an elastic blister in the presence of sloping topography. *J. Fluid Mech.* **967**, A5 (2023).
10. Stevens, L. A., Hewitt, I. J., Das, S. B. & Behn, M. D. Relationship Between Greenland Ice Sheet Surface Speed and Modeled Effective Pressure. *J. Geophys. Res. Earth Surf.* **123**, 2258–2278 (2018).
11. Chu, W., Creyts, T. T. & Bell, R. E. Rerouting of subglacial water flow between neighboring glaciers in West Greenland. *J. Geophys. Res. Earth Surf.* **121**, 925–938 (2016).
12. Andrews, L. C. *et al.* Seasonal Evolution of the Subglacial Hydrologic System Modified by Supraglacial Lake Drainage in Western Greenland. *J. Geophys. Res. Earth Surf.* **123**, 1479–1496 (2018).
13. Hooke, R. LeB. Englacial and Subglacial Hydrology: A Qualitative Review. *Arct. Alp. Res.* **21**, 221–233 (1989).
14. Blake, W., Fischer, U. H., Bentley, C. R. & Clarke, G. K. G. Instruments and Methods: Direct measurement of sliding at the glacier bed. *J. Glaciol.* **40**, 595–599 (1994).
15. Morlighem, M. & Et Al. IceBridge BedMachine Greenland, Version 5. NASA National Snow and Ice Data Center Distributed Active Archive Center https://doi.org/10.5067/GMEVBWFLWA7X (2022).
16. Morlighem, M. *et al.* BedMachine v3: Complete Bed Topography and Ocean Bathymetry Mapping of Greenland From Multibeam Echo Sounding Combined With Mass Conservation. *Geophys. Res. Lett.* **44**, (2017).
17. Noël, B., Van De Berg, W. J., Lhermitte, S. & Van Den Broeke, M. R. Rapid ablation zone expansion amplifies north Greenland mass loss. *Sci. Adv.* **5**, eaaw0123 (2019).
18. Stevens, L. A. *et al.* Greenland supraglacial lake drainages triggered by hydrologically induced basal slip. *Nature* **522**, 73–76 (2015).
