# Supplementary material for: Ice-sheet hydro-fracture not advanced inland by lower-elevation lake drainages in Kalaallit Nunaat
Source: Nat Commun. 2026 May 27;17:4598. doi: 10.1038/s41467-026-73033-z (PMC13216594; doi:10.1038/s41467-026-73033-z)
Supplement: Supplementary file 2 — Description of Additional Supplementary Files [file 41467_2026_73033_MOESM2_ESM.pdf]

Description of Additional Supplementary Files

File name: Supplementary Movie 1

Description: **Surface runoff, subglacial discharge, and lake-drainage events in 2022. (upper panel)** (blue colormap) Daily surface runoff for study region estimated by distributing RACMO runoff estimates to 300-m-spaced model grid nodes. Day of year of movie frame plotted as map title. (**middle panel**) (blue colormap) Modelled subglacial discharge q forced by the daily, distributed surface runoff inputs shown in upper panel. The subglacial-discharge colormap is truncated such that values below 10^{-4} m^2 s^{-1} plot as white. In both map-view panels, ice-sheet surface elevation shown with 100-m-elevation contours in grey. GNSS stations shown with solid black triangles. Black-outlined symbols show location and drainage mechanism of supraglacial lakes, with the symbol color indicating whether the lake is (blue) filling; (goldenrod) initially draining; (green) continuing to drain; or (grey) an empty, dry, or frozen lake basin. Lake-drainage mechanisms are: (stars) hydro-fracture, (overturned triangles) moulin, (circles) overspill, and (square) no-exit, frozen. The location and drainage timing of (diamonds) water-filled crevasses are also shown. Lakes and water-filled crevasses are first plotted in time on the day of year in which they attain v_{crit}, their critical volume required to hydro-fracture to the ice-sheet bed (Methods). (**bottom panel**) (solid-blue line) Surface-runoff and (dashed-blue line) basal-melt inputs summed across the model domain; proglacial discharge exiting the model domain shown in yellow. Purple vertical bar tracks time.

File name: Supplementary Movie 2

Description: **Surface runoff, subglacial discharge, and lake-drainage events in 2023.** Equivalent to Supplementary Movie 1, but for 2023. **(upper panel)** (blue colormap) Daily surface runoff for study region estimated by distributing RACMO runoff estimates to 300-m-spaced model grid nodes. Day of year of movie frame plotted as map title. (**middle panel**) (blue colormap) Modelled subglacial discharge q forced by the daily, distributed surface runoff inputs shown in upper panel. The subglacial-discharge colormap is truncated such that values below 10^{-4} m^2 s^{-1} plot as white. In both map-view panels, ice-sheet surface elevation shown with 100-m-elevation contours in grey. GNSS stations shown with solid black triangles. Black-outlined symbols show location and drainage mechanism of supraglacial lakes, with the symbol color indicating whether the lake is (blue) filling; (goldenrod) initially draining; (green) continuing to drain; or (grey) an empty, dry, or frozen lake basin. Lake-drainage mechanisms are: (stars) hydro-fracture, (overturned triangles) moulin, (circles) overspill, and (square) no-exit, frozen. The location and drainage timing of (diamonds) water-filled crevasses are also shown. Lakes and water-filled crevasses are first plotted in time on the day of year in which they attain v_{crit}, their critical volume required to hydro-fracture to the ice-sheet bed (Methods). (**bottom panel**) (solid-blue line) Surface-runoff and (dashed-blue line) basal-melt inputs summed across the model domain; proglacial discharge exiting the model domain shown in yellow. Purple vertical bar tracks time.
